# Oxidative phosphorylation is required for cardiomyocyte re-differentiation and long-term fish heart regeneration

**DOI:** 10.1038/s44161-025-00718-x

**Published:** 2025-10-01

**Authors:** Konstantinos Lekkos, Zhilian Hu, Phong D. Nguyen, Hessel Honkoop, Esra Sengul, Rita Alonaizan, Jana Koth, Jun Ying, Madeleine E. Lemieux, Alisha Kenward, Sean Keeley, Bastiaan Spanjaard, Brett W. C. Kennedy, Xin Sun, Katherine Banecki, Helen G. Potts, Gennaro Ruggiero, James Montgomery, Daniela Panáková, Jan Philipp Junker, Lisa C. Heather, Xiaonan Wang, Juan Manuel Gonzalez-Rosa, Jeroen Bakkers, Mathilda T. M. Mommersteeg

**Affiliations:** 1 Institute of Developmental and Regenerative Medicine, University of Oxford, Oxford, UK; 2 Department of Physiology, Anatomy & Genetics, University of Oxford, Oxford, UK; 3 Hubrecht Institute-KNAW and University Medical Center Utrecht, Utrecht, The Netherlands; 4 Institut Curie, PSL University, Sorbonne Université, CNRS UMR3215, Inserm U934, Genetics and Developmental Biology, Paris, France; 5 King Faisal Specialist Hospital & Research Centre, Riyadh, Saudi Arabia; 6 MRC Molecular Haematology Unit, Weatherall Institute of Molecular Medicine, John Radcliffe Hospital, University of Oxford, Oxford, UK; 7 Faculty of Medical Laboratory Science, College of Health Science and Technology, Shanghai Jiao Tong University School of Medicine, Shanghai, China; 8 Bioinfo, Plantagenet, Ontario Canada; 9 Department of Biology, Morrissey College of Arts and Sciences, Boston College, Chestnut Hill, MA USA; 10 Max Delbrück Center for Molecular Medicine, Berlin, Germany; 11 Charité – Universitätsmedizin Berlin, Berlin, Germany; 12 Department of Chemistry, University of Oxford, Oxford, UK; 13 DZHK (German Centre for Cardiovascular Research) partner site, Berlin, Germany; 14 Department of Congenital Heart Disease and Pediatric Cardiology, University Hospital Schleswig-Holstein, Kiel, Germany; 15 School of Public Health, Shanghai Jiao Tong University School of Medicine, Shanghai, China; 16 Cardiovascular Research Center, Massachusetts General Hospital Research Institute and Harvard Medical School, Boston, MA USA; 17 Department of Pediatric Cardiology, Division of Pediatrics, University Medical Center Utrecht, Utrecht, The Netherlands

**Keywords:** Regeneration, Heart stem cells

## Abstract

In contrast to humans, fish can fully regenerate their hearts after cardiac injury. However, not all fish have the same regenerative potential, allowing comparative inter-species and intra-species analysis to identify the mechanisms controlling successful heart regeneration. Here we report a differential regenerative response to cardiac cryo-injury among different wild-type zebrafish strains. Correlating these data with single-cell and bulk RNA sequencing data, we identify oxidative phosphorylation (OXPHOS) as a positive regulator of long-term regenerative outcome. OXPHOS levels, driven by glycolysis through the malate-aspartate shuttle, increase as soon as cardiomyocyte proliferation decreases, and this increase is required for cardiomyocyte re-differentiation and successful long-term regeneration. Reduced upregulation of OXPHOS in *Astyanax mexicanus* cavefish results in the absence of a dynamic temporal sarcomere gene expression program during cardiomyocyte re-differentiation. These findings challenge the assumption that OXPHOS inhibits regeneration and reveal targetable pathways to enhance heart repair in humans after myocardial infarction.

## Abstract (web summary)

Lekkos et al. show that a metabolic switch toward oxidative phosphorylation is required for cardiomyocyte re-differentiation and heart regeneration after injury in fish.

## Main

The ability for heart regeneration during adult life varies considerably among species. Mammals, including humans, have very limited regenerative capacity in the heart, whereas other species, including zebrafish, can completely restore damaged tissue^1,2^. The current stance in the field is that this difference is underpinned by species-specific variations in cardiomyocyte metabolism^3,4^. After birth, mammals are exposed to an oxygen-rich environment in comparison to the more hypoxic conditions in utero, resulting in a switch from a glycolytic metabolism to OXPHOS largely fueled by fatty acid β-oxidation. This allows cardiomyocytes to meet the increased demand for cardiac output in warm-blooded species^5^ but also generates reactive oxygen species (ROS), which can damage DNA. As a result, cardiomyocytes that were highly proliferative before birth lose this ability after the first weeks of life^6,7^. By contrast, fish live in water that has lower levels of oxygen than the surrounding air, which is thought to facilitate glycolytic metabolism throughout adulthood. Cold-blooded fish have lower cardiac output, and it has been suggested that the cardiomyocytes are less specialized; therefore, they do not require the high levels of ATP produced by OXPHOS and do not compromise on proliferative capacity. By using glycolysis, zebrafish cardiomyocytes can pass their cell cycle checkpoints and divide, restoring the lost myocardium after injury^7–12^.

Numerous findings have confirmed the beneficial role of glycolysis during heart regeneration in fish. Glycolysis is strongly upregulated in the cardiomyocytes bordering the injured area, and, when inhibited, cardiomyocyte proliferation is reduced^13^. Additionally, uncoupling glycolysis from pyruvate oxidation, by preventing glucose from shunting toward OXPHOS through overexpression of *pdk* (pyruvate dehydrogenase kinase), increases cardiomyocyte proliferation^14^. However, the role of oxidative metabolism in the regenerative process remains elusive even though some studies have hinted that there is an upregulation of *OXPHOS* during heart regeneration^15–17^. The reported increase in *OXPHOS* at 7 days post-cryo-injury (dpci), which coincides with the peak of cardiomyocyte proliferation^18^, seems counterintuitive and leaves many open questions about its role in regeneration.

Here, with an intra-species and inter-species comparative approach using zebrafish and *A. mexicanus*, we show that, during successful regeneration, *OXPHOS* is downregulated in the first days after injury while cardiomyocyte proliferation takes place. However, immediately after the peak of proliferation, the *malate-aspartate shuttle (MAS)* is upregulated, activating the *tricarboxylic acid (TCA)* cycle and *OXPHOS*. The upregulation of *OXPHOS* coincides with a dynamic pattern of re-differentiation of the border zone cardiomyocytes and determines the long-term regenerative outcome. Pharmacologically or genetically blocking the MAS or OXPHOS inhibits cardiomyocyte re-differentiation and regeneration. Therefore, the view on zebrafish heart regeneration as being solely reliant on glycolysis, with OXPHOS seen as exclusively detrimental, needs revisiting.

## Results

### Differential regenerative response among wild-type zebrafish strains

**Fig. 1:**
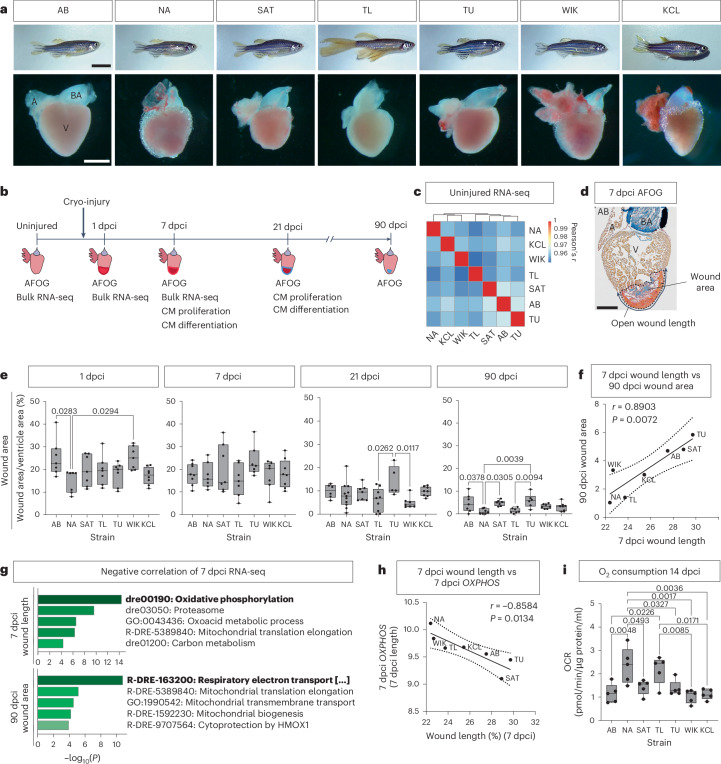
Differential regenerative response to cryo-injury among wild-type adult zebrafish strains identifies OXPHOS as beneficial for regeneration. **a**, Representative images of adult wild-type zebrafish strains (scale bar, 1 cm) and their intact heart, including atrium (A), bulbus arteriosus (BA) and ventricle (V) (scale bar, 500 μm). **b**, Experimental procedure to harvest and assay zebrafish hearts prior to and after cryo-injury. **c**, Correlation matrix of bulk RNA-seq data on uninjured ventricles showing a strong correlation between strains. **d**, Representative AFOG image of an AB heart at 7 dpci indicating wound area and open wound length measurements (scale bar, 300 µm). **e**, Wound area quantification of the seven wild-type strains showed significant differences at 1, 21 and 90 dpci among the strains. **f**, Positive correlation between 7 dpci wound length and 90 dpci wound area. **g**, The top five enriched processes in the genes negatively correlating to wound length at 7 dpci and area at 90 dpci in the 7 dpci bulk RNA-seq. **h**, Significant negative correlation between 7 dpci wound length and *OXPHOS*. **i**, OCR measurements of 14 dpci ventricles show significant differences in OXPHOS among the seven wild-type zebrafish strains. **c**, *n* = 3 biological replicates per zebrafish strain; **e**, 1 dpci: AB, NA, SAT, TL, TU, WIK *n* = 7; KCL *n* = 8. 7 dpci: NA, SAT, TL, TU, WIK *n* = 7; AB, KCL *n* = 8. 21 dpci: SAT, WIK *n* = 7; AB *n* = 6; NA *n* = 11; TL *n* = 9; TU *n* = 5; KCL *n* = 8. 90 dpci: AB, NA, SAT, TL, WIK *n* = 7; TU *n* = 6; KCL *n* = 8 (biological replicates). **i**, *n* = 5 biological replicates per strain. **e**,**i**, One-way ANOVA with Tukey’s test. **f**–**h**, Simple linear regression. **g**, Analysis performed using Metascape. CM, cardiomyocyte. Source data

**Extended Data Fig. 1:**
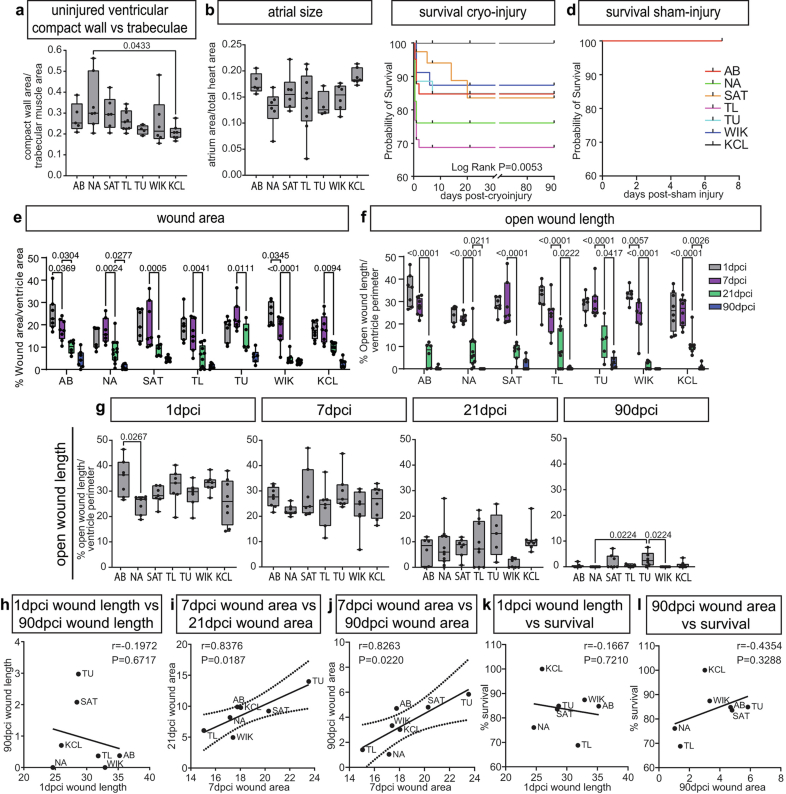
Differential regenerative response to cryoinjury between wild-type adult zebrafish strains. (**a**) Ratio of ventricular compact wall area and trabecular area as indication of sponginess of the hearts showing differences trabeculae between adult NA and KCL but not between other strains. (**b**) Quantification of atrial size showing no differences between the strains. (**c**) Survival curve until 90dpci showing differences in survival between the strains. (**d**) Survival curve until 7 days post sham. (**e-f**) Quantification of wound area (**e**) and length (**f**) over time in all the strains (only significant p-value between adjacent time points per strain is shown). (**g**) Wound length quantification showing significant differences at 1 and 90dpci between the strains. (**h**) No correlation between 1dpci wound length and 90dpci wound length. (**i-j**) Positive correlation between 7- and 21dpci (**i**) and 21- and 90dpci wound area (**j**). (**k**) No correlation between extend of injury (1dpci wound length) and survival. (**l**) No correlation between long-term regeneration (90dpci wound area) and survival. **a**, AB n=5; NA, KCL n=7; SAT, WIK n=6; TL n=9; TU n=4 (biological replicates); **b**, AB n=5; NA, SAT, WIK n=6; TL n=9; TU n=4; KCL n=7 (biological replicates); **c**, AB n=42, NA n=46, SAT n=38, TL n=52, TU n=35, WIK n=34, KCL n=32 (biological replicates); **d**, n=5 biological replicates per strain; **e**, AB: 1, 90dpci n=7; 7dpci n=8; 21dpci n=6. NA: 1, 7, 90dpci n=7; 21dpci n=11. SAT: 1, 7, 21, 90dpci n=7. TL: 1, 7, 90dpci n=7; 21dpci n=9. TU: 1, 7dpci n=7; 21dpci n=5; 90dpci n=6. WIK: 1, 7, 21, 90dpci n=7. KCL: 1, 7, 21, 90dpci n=8 (biological replicates); **f**, AB: 1, 90dpci n=7; 7dpci n=8; 21dpci n=6. NA: 1, 7, 90dpci n=7; 21dpci n=11. SAT: 1, 7, 21, 90dpci n=7. TL: 1, 7, 90dpci n=7; 21dpci n=9. TU: 1, 7dpci n=7; 21dpci n=5; 90dpci n=6. WIK: 1, 7, 21, 90dpci n=7. KCL: 1, 7, 21, 90dpci n=8 (biological replicates); **g**, 1dpci: AB, NA, SAT, TL, TU, WIK n=7; KCL n=8. 7dpci: NA, SAT, TL, TU, WIK n=7; AB, KCL n=8. 21dpci: SAT, WIK n=7; AB n=6; NA n=11; TL n=9; TU n=5; KCL n=8. 90dpci: AB, NA, SAT, TL, WIK n=7; TU n=6; KCL n=8 (biological replicates). **a**, **b**, **g**, one-way ANOVA with Tukey’s test; **c**, **d**, Kaplan-Meier assay with Log-rank (Mantel-Cox) test; **e**, **f**, two-way ANOVA with Šídák’s multiple test; **h**-**l**, simple linear regression. Source data

Although adult zebrafish can repair damaged heart muscle in response to cryo-injury, the speed of regeneration varies among studies^19–25^. Those differences can be attributed to inter-study deviations in surgery techniques. However, the background strain used also varies, indicating possible differential responses to cryo-injury among strains. To identify potential intra-species variabilities in cardiac regeneration, we investigated the response of seven different wild-type zebrafish strains to cryo-injury. In addition to the strain already used in our facility (Kings College London (KCL)), we imported six zebrafish strains: AB, Nadia (NA), Sanger AB Tübingen (SAT), Tupfel long fin (TL), Tübingen (TU) and Wild India Kolkata (WIK) (Fig. 1a). Hearts were isolated at 1, 7, 21 and 90 dpci as well as uninjured controls and sham (Fig. 1a,b). Analysis of the uninjured hearts showed strong similarity among the strains but with some differences in morphology and transcriptome (Fig. 1a,c and Extended Data Fig. 1a,b). Despite the overall similarities, post-cryo-injury survival was significantly different among the strains (Extended Data Fig. 1c,d). To assess differences in regenerative response, we quantified the wound area and open wound length in all strains over time (Fig. 1d,e and Extended Data Fig. 1e–g). At 1 dpci, NA had significantly smaller wounds compared to other strains. At 7 dpci, no significant differences were observed; however, at 21 dpci, TU showed significantly larger wound area. At 90 dpci, when the cryo-injury-induced wound is reported to be strongly reduced or to have disappeared^20,26^, there were large differences among the strains. TU and SAT had the largest wound area/length, whereas NA and TL had the smallest wounds (Fig. 1e and Extended Data Fig. 1e–g). Comparing the data between the different timepoints showed positive correlation among 7 dcpi, 21 dcpi and 90 dpci wound length/area but not 1 dpci (Fig. 1f and Extended Data Fig. 1h–j), indicating that any experimental variation in cryo-injury did not influence end-stage regeneration. There was also no correlation between survival and the extent of injury (Extended Data Fig. 1k,l). These data indicate that the response to cryo-injury is heterogeneous among different wild-type zebrafish strains.

### OXPHOS is beneficial for heart regeneration

**Extended Data Fig. 2:**
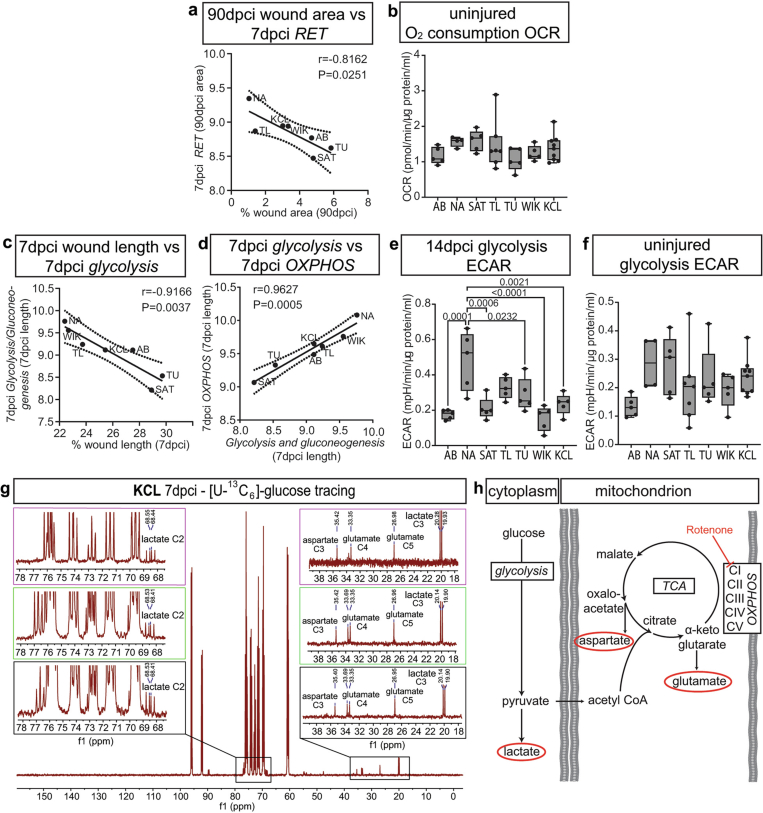
Oxidative phosphorylation is beneficial for regeneration. (**a**) Negative correlation between 90dpci wound area and the 7dpci Respiratory electron transport GO-term. (**b**) Oxygen consumption rate (OCR) measurements of uninjured ventricles show similar levels of OXPHOS among the seven wild-type zebrafish strains. (**c**) Negative correlation between 7dpci wound length and 7dpci Glycolysis. (**d**) Significant positive correlation between Glycolysis and OXPHOS at 7dpci. (**e**) Extracellular acidification rate (ECAR) of 14dpci ventricles show significant differences in glycolysis among the seven wild-type zebrafish strains. (**f**) Extracellular acidification rate (ECAR) measurements of uninjured ventricles show similar levels of glycolysis among the seven wild-type zebrafish strains. (**g**) Nuclear magnetic resonance (NMR) analysis of 13C-glucose labelled 7dpci KCL hearts identified glutamate, aspartate and lactate as metabolites (representative spectrum and zoomed-in panels in black boxes, additional biological replicas in green and magenta boxes, total number of replicas n=3 containing 10 pooled hearts each). (**h**) Schematic of NMR traced [U-13C6]-glucose being converted into lactate or entering the tricarboxylic acid (TCA) cycle via pyruvate to generate glutamate and aspartate. **b**, **f**, AB, SAT, TU, WIK n=5; NA n=4; TL n=7; KCL n=9 (biological replicates); **e**, n=5 biological replicates per strain; **g**. n=3 replicates containing 10 pooled hearts each. **a**, **c**-**d**, simple linear regression; **b**, **e**-**f**, one-way ANOVA with Tukey’s test. RET, Respiratory Electron Transport chain; Rotenone, as used in Fig. 2c, inhibits OXPHOS through its action on Complex I; C2-4, indicates the number of the carbon labelled by 13C; CI-CV, complex I-V. Source data

To investigate whether the observed differences might uncover mechanisms underlying regeneration, we analyzed the 7 dpci RNA sequencing (RNA-seq) data to identify genes and enriched processes significantly correlating to wound area/length (Pearsonʼs *r* > 0.75 or *r* < −0.75)^26^. Unexpectedly, this identified *Oxidative* *phosphorylation* and *Respiratory* *transport*, *ATP* *synthesis* *by* *chemiosmotic* *coupling* *and* *heat* *production* *by* *uncoupling* *protein* (further referred to as *OXPHOS*) as the top enriched processes negatively correlating with 7 dpci wound length and 90 dpci wound area, respectively, suggesting that upregulated OXPHOS may promote cardiac regeneration (Fig. 1g,h, Extended Data Fig. 2a and Supplementary Tables 1 and 2). Oxygen consumption rate (OCR) measurements confirmed the functional upregulation of OXPHOS in the best regenerating strains at 14 dpci (Fig. 1i) but not before injury (Extended Data Fig. 2b). *Glycolysis and gluconeogenesis* (further referred to as *Glycolysis*) was also enriched in the genes negatively correlating to 7 dpci (subcategory of *Carbon metabolism*; Extended Data Fig. 2c and Supplementary Table 1) and correlated strongly with *OXPHOS* (Extended Data Fig. 2d), indicating that these processes are linked. Indeed, extracellular acidification rate (ECAR), a functional measurement of glycolytic flux, was highest in 14 dpci NA and TL with no differences before injury (Extended Data Fig. 2e,f). Identification of glutamate and lactate derived from the exogenous [U-^13^C_6_]-glucose confirms that glucose is both anaerobically metabolized to lactate and aerobically oxidized within the TCA in 7 dpci KCL ventricles (Extended Data Fig. 2g,h). These data suggest a previously unidentified beneficial role for OXPHOS, driven by glucose oxidation, during heart regeneration.

### Upregulation of the MAS drives OXPHOS and heart regeneration

**Fig. 2:**
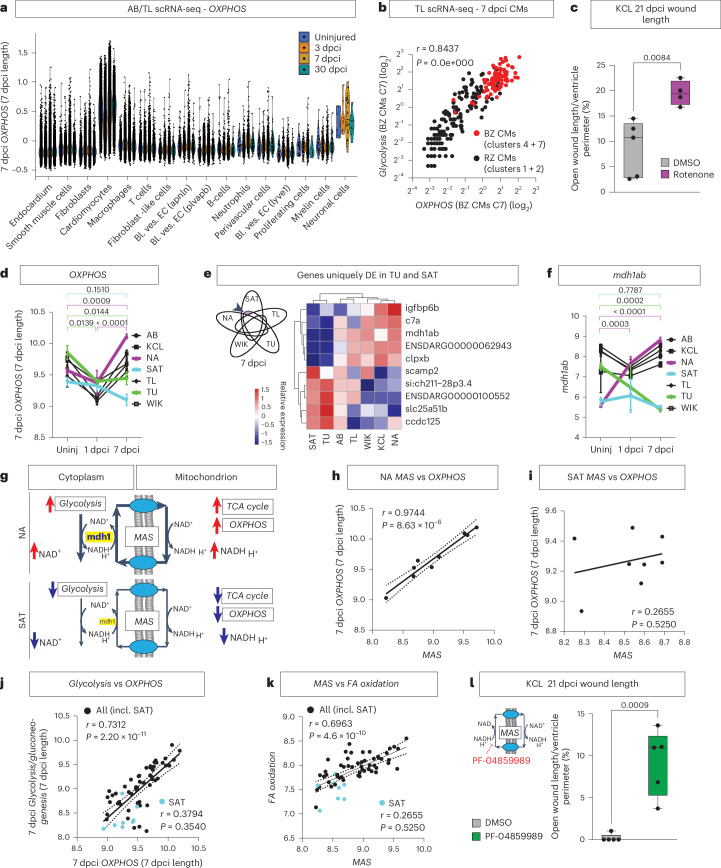
Upregulation of the MAS drives *OXPHOS* and regeneration. **a**, *OXPHOS* gene expression is highest in cardiomyocytes compared to other cell types in scRNA-seq of AB/TL—reanalysis of Hu et al.^27^. **b**, Positive correlation between *Glycolysis* and *OXPHOS* in 7 dpci cardiomyocytes, with highest levels of both in the border zone cardiomyocytes (red), by reanalyzing scRNA-seq data of TL published previously by Honkoop et al.^13^. Clusters 1, 2, 4 and 7 refer to the clustering in the original publication. **c**, Increased wound length in KCL treated with rotenone in comparison to DMSO control at 21 dpci. **d**, Temporal pattern of *OXPHOS* gene expression in the bulk RNA-seq data. **e**, Venn diagram indicating uniquely differentially expressed (DE) genes in TU and SAT (AB and KCL not shown but included in analysis) with heatmap of the top DE genes in the bulk RNA-seq identifying *mdh1ab* uniquely downregulated in TU and SAT versus the other strains. **f**, Expression pattern of *mdh1ab* over time in the bulk RNA-seq data of the strains. **g**, Schematic diagram indicating the influence of activated MAS on maintaining cytosolic and mitochondrial balance of NAD^+^/NADH in NA versus SAT. **h**,**i**, Positive correlation between the *MAS* and *OXPHOS* in the NA strain (**h**) but not the SAT strain (**i**). **j**,**k**, Positive correlation of *Glycolysis* with *OXPHOS* (**j**) and *MAS* versus *Fatty Acid* (*FA*) *oxidation* (**k**) in all strains but absence of correlation in SAT. **l**, Wound length quantification showing increased 21 dpci wound length in KCL hearts treated with MAS inhibitor PF-04859989 in comparison to DMSO control. **c**, Rotenone *n* = 4, DMSO *n* = 5 (biological replicates); **d**,**f**, uninjured: *n* = 3 per strain, 1 dpci: SAT, TU *n* = 2, AB, NA, TL, WIK, KCL *n* = 3, 7 dpci *n* = 3 per strain (biological replicates); **e**, *n* = 3 biological replicates per strain; **l**, *n* = 5 biological replicates per group. **b**,**h**–**k**, Simple linear regression. **c**,**l**, Unpaired two-tailed Student’s *t*-test. **d**,**f**, Two-way ANOVA with Tukey’s test; data presented as mean ± s.e.m. Bl. ves. EC, blood vessel endothelial cell; BZ, border zone; C, cluster; CM, cardiomyocyte; incl., including; RZ, remote zone; Uninj, uninjured. Source data

**Extended Data Fig. 3:**
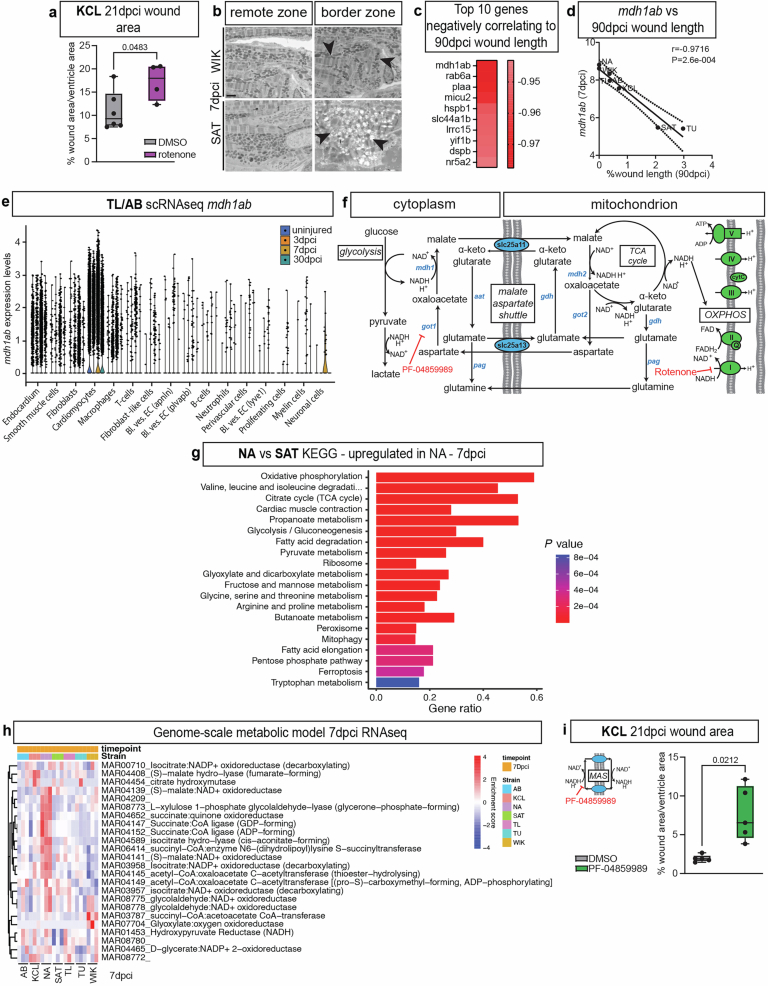
Activation of the Malate Aspartate Shuttle drives OXPHOS and regeneration. (**a**) Increased wound area in KCL treated with rotenone in comparison with DMSO control at 21dpci. (**b**) Representative scanning electron microscope images displaying mitochondrial morphology in the border zone versus remote zone cardiomyocytes in WIK and SAT strains at 7dpci. Arrow heads point to different morphology of border zone mitochondria between WIK and SAT. Scale bar 2µm. (**c**) Heatmap of top 10 genes expressed at 7dpci in the RNAseq negatively correlating to 90dpci wound length, with mdh1ab as top gene. (**d**) Negative correlation of 7dpci mdh1ab expression to wound length at 90dpci. (**e**) Violin plots showing high mdh1ab gene expression in cardiomyocytes compared to other cell types in re-analysed scRNAseq data of AB/TL strains at 3-, 7- and 30dpci as well as uninjured hearts. (**f**) Schematic diagram showing the malate aspartate shuttle (MAS) in relation to glycolysis, the TCA cycle and OXPHOS with inhibitors used in red letters. (**g**) KEGG pathway enrichment analysis of the upregulated genes in NA 7dpci compared to SAT 7dpci, with fold change (FC) > 1.5 and false discovery rate (FDR) <0.1. (**h**) Reaction enrichment scores of metabolic pathways within the TCA subset of the zebrafish genome-scale metabolism model (GEM). (**i**) Wound area quantification showing increased 21dpci wound area in KCL hearts treated with inhibitor PF-04859989 in comparison to DMSO control. **a**, rotenone n=4, DMSO n=6 (biological replicates); **i**, n=5 biological replicates per group. **a**, unpaired two-tailed Student’s t-test; **c**-**d**, simple linear regression; **g**, Fisher exact test with Benjamini-Hochberg method for multiple hypotheses testing; **h** scaled enrichment score calculated from raw gene expression counts by troppo, with default parameters; **i**, unpaired two-tailed Welch t-test. Source data

By reanalyzing published single-cell RNA sequencing (scRNA-seq) datasets from Hu et al.^27^ and Honkoop et al.^13^, we confirmed that *OXPHOS* was highest in border zone cardiomyocytes (Fig. 2a,b)^13^. To test whether OXPHOS is indeed beneficial for regeneration, we treated KCL fish with rotenone, which inhibits complex I of the respiratory electron transport chain (ETC). Rotenone treatment resulted in larger wounds at 21 dpci compared to control DMSO-treated fish (Fig. 2c and Extended Data Fig. 3a). These data indicate that upregulation of OXPHOS in border zone cardiomyocytes is not detrimental to regeneration, as previously thought, and may be required for optimal regeneration.

To identify the mechanism underlying the upregulation of *OXPHOS*, we plotted *OXPHOS* over time in the different strains (Fig. 2d). This showed that, after an initial dip at 1 dpci, in contrast to the other strains, *OXPHOS* levels did not recover at 7 dpci in TU and SAT (largest 90 dpci wounds). To analyze if this lack of recovery was due to mitochondrial dysfunction, we performed electron microscopy of cardiomyocytes in SAT and WIK. Although mitochondria in WIK showed a similar morphology in both border zone and remote zone cardiomyocytes, the mitochondria in SAT were more swollen and disorganized in the border zone compared to the remote zone, which are hallmarks of mitochondrial failure (Extended Data Fig. 3b)^13,28^. Plotting the genes uniquely upregulated or downregulated in both SAT and TU strains at 7 dpci identified the cytoplasmic malate dehydrogenase gene *mdh1ab*, which was also the top gene negatively correlating to 90 dpci wound length (Fig. 2e,f and Extended Data Fig. 3c,d). scRNA-seq data confirmed *mdh1ab* to be mainly expressed in cardiomyocytes (Extended Data Fig. 3e).

Mdh1 is important for maintaining the balance of NAD^+^/NADH between the cytosol and the mitochondria as part of the MAS (Extended Data Fig. 3f)^29^. High levels of MAS activity (as in NA) allow for recycling of NAD^+^ in the cytosol, which can feed back into glycolysis with simultaneous regeneration of NADH in the mitochondria fueling the TCA cycle and OXPHOS. Therefore, although NAD^+^ can also be replenished by lactate secretion under anaerobic conditions, the net result of low levels of MAS (as in SAT) could result in less glycolysis as well as less OXPHOS (Figs. 1i and 2g and Extended Data Fig. 2d,e). In line with this, *MAS* levels correlated with levels of *OXPHOS* in the highly regenerative NA, but this correlation was absent in the poorly regenerating SAT strain (Fig. 2h,i). Moreover, although *Glycolysis* generally correlated with *OXPHOS*, this was disrupted in the SAT strain (Fig. 2j). As the MAS provides malate into the second span of the TCA cycle, a source of acetyl CoA is simultaneously required for TCA cycling. Although we showed that this acetyl CoA can be supplied by glucose, this does not exclude contribution from fatty acid β-oxidation. Indeed, we found that *MAS* gene expression levels also correlated with the levels of fatty acid β-oxidation, and this was again absent in the SAT (Fig. 2k). This indicates that, although the MAS is driven by glycolysis, its activation of the TCA cycle allows for a general increase in mitochondrial metabolism, including activation of biosynthetic pathways, which we confirmed by comparing NA and SAT using Kyoto Encyclopedia of Genes and Genomes (KEGG) pathway analysis and genetic-scale metabolic modeling (Extended Data Fig. 3g,h and Supplementary Tables 3 and 4). To confirm the importance of the MAS for regeneration, we exposed KCL fish to the small-molecule inhibitor PF-04859989 to block the MAS^30^, which resulted in reduced regeneration compared to DMSO-treated controls (Fig. 2i and Extended Data Fig. 3f,i). These data indicate that glycolysis and mitochondrial metabolism are tightly coupled via the MAS and that activation of the MAS is required for regeneration.

### OXPHOS is not required for cardiomyocyte proliferation

**Fig. 3:**
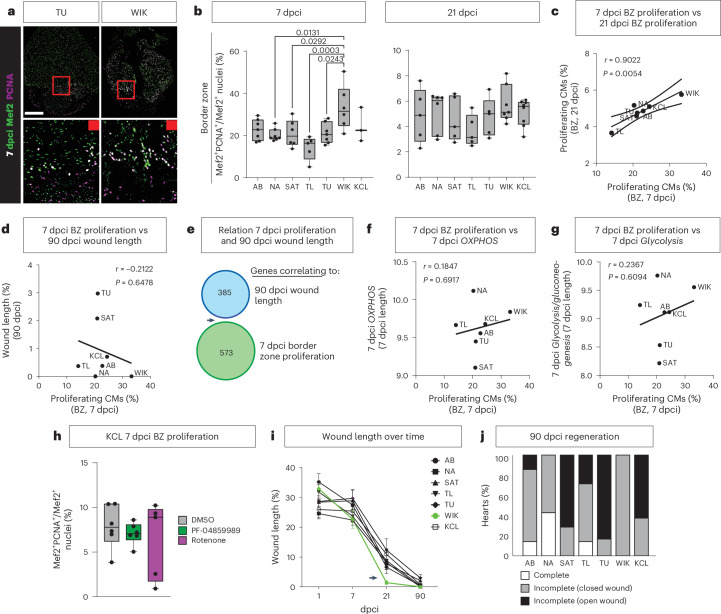
OXPHOS is not required for cardiomyocyte proliferation. **a**, Representative images of immunofluorescence staining showing Mef2^+^PCNA^+^ double-positive proliferating cardiomyocytes in TU and WIK at 7 dpci. Framed areas highlight the wound border zone (red) (scale bar, 300 μm). **b**, Quantification of Mef2^+^PCNA^+^ double-positive cells showing differences in proliferating cardiomyocytes in the border zone at 7 dpci but not at 21 dpci. **c**, Positive correlation of percentage of proliferating cardiomyocytes in the border zone between 7 dpci and 21 dpci. **d**, No correlation between 7 dpci border zone proliferation and 90 dpci wound length. **e**, Venn diagram displaying complete lack of overlap between genes correlating to 7 dpci border zone proliferation and 90-dpci wound length. **f**,**g**, No correlation between border zone proliferation and *OXPHOS* (**f**) or *Glycolysis* (**g**) at 7 dpci. **h**, Quantification of Mef2^+^PCNA^+^ cells showing no difference in proliferating cardiomyocytes in the border zone of 7 dpci KCL adult treated with inhibitor PF-04859989 or rotenone compared to DMSO control. **i**, Temporal wound length reduction of all strains between 1, 7, 21 and 90 dpci. Arrow highlighting the strong decrease in wound length in WIK between 7 dcpi and 21 dpci. **j**, Percentage of hearts completely regenerated at 90 dpci or with closed compact wall but remaining internal scar or with open compact wall and internal scar remaining. **b**, 7 dpci: AB, NA, TU *n* = 7; SAT, WIK *n* = 6; TL *n* = 5; KCL *n* = 3, 21 dpci: AB, SAT, TL, TU *n* = 5; NA *n* = 6; WIK *n* = 7; KCL *n* = 8 (biological replicates); **h**, PF-04859989, DMSO *n* = 6, rotenone *n* = 5 (biological replicates); **i**, AB: 1, 90 dpci *n* = 7; 7 dpci *n* = 8; 21 dpci *n* = 6. NA: 1, 7, 90 dpci *n* = 7; 21 dpci *n* = 11. SAT: 1, 7, 21, 90 dpci *n* = 7. TL: 1, 7, 90 dpci *n* = 7; 21 dpci *n* = 9. TU: 1, 7 dpci *n* = 7; 21 dpci *n* = 5; 90 dpci *n* = 6. WIK: 1, 7, 21, 90 dpci *n* = 7. KCL: 1, 7, 21, 90 dpci *n* = 8 (biological replicates); **j**, AB, NA, SAT, TL, WIK *n* = 7; TU *n* = 6; KCL *n* = 8. **b**,**h**, One-way ANOVA with Tukey’s test. **c**,**d**,**f**,**g**, Simple linear regression. **h**, Two-way ANOVA with Tukey’s test. **i**, Data presented as mean ± s.e.m. Mef2, myocyte enhancer factor 2; PCNA, proliferating cell nuclear antigen. Source data

**Extended Data Fig. 4:**
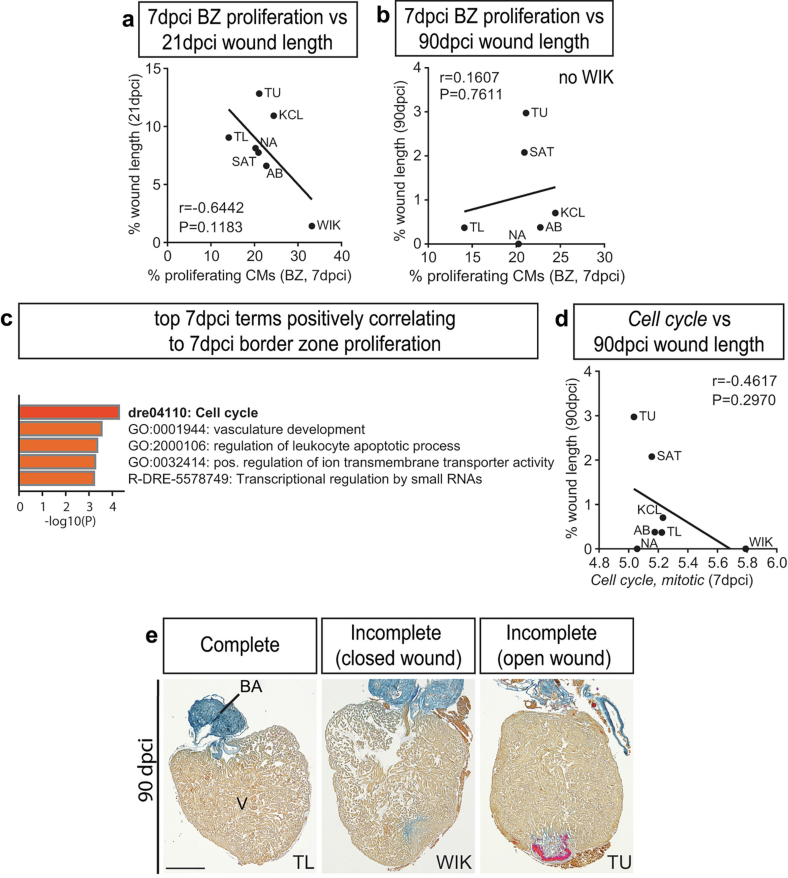
OXPHOS is not required for cardiomyocyte proliferation. (**a-b**) No correlation between 7dpci border zone proliferation and 90dpci wound length in all strains (**a**) or those excluding WIK (**b**). (**c**) The top 5 enriched processes in the genes positively correlating to 7dpci border zone proliferation in the 7dpci bulk RNAseq. (**d**) No correlation between 7dpci cell cycle genes and 90dpci wound length. (**e**) Representative AFOG images of cryo-injured hearts at 90dpci to indicate classification (Scale bar: 300µm). **d**, measurements of those two parameters result from two independent experiments that were performed once (the data results from different timepoints and techniques and is not paired, consequently the average measurements per characteristic have been used). **a-b, d**, simple linear regression; **c**, analysis performed using Metascape.

The established increase in *OXPHOS* in regenerative border zone cardiomyocytes seems paradoxical as OXPHOS is thought to cause cell cycle arrest^7^. To investigate this, we quantified cardiomyocyte proliferation across the strains at 7 dcpi and 21 dpci. The reported peak of cardiomyocyte proliferation is 7 dpci, which declines toward baseline by 21 dpci^31^. At 7 dpci, only WIK had significantly more proliferating cardiomyocytes in the border zone myocardium compared to the other strains. At 21 dpci, proliferation had strongly reduced, and this difference was absent (Fig. 3a,b). TL, one of the best regenerating strains at 90 dpci, had the lowest percentage of proliferation at both 7 dcpi and 21 dpci (Fig. 3b). Levels of border zone proliferation correlated between 7 dcpi and 21 dpci (Fig. 3c). Surprisingly, however, 7 dpci border zone proliferation did not correlate to 21 dcpi or 90 dpci wound size (Fig. 3d and Extended Data Fig. 4a). Removing the ‘outlier’ WIK did not influence these results, indicating that the lack of correlation with 90 dpci wound size was not solely driven by the WIK measurements (Extended Data Fig. 4b). Correspondingly, there was no overlap between the genes correlating to border zone proliferation and 90 dpci wound size (Fig. 3e, arrowhead; Extended Data Fig. 4c,d; and Supplementary Table 5), and no correlation between proliferation and levels of *OXPHOS* was observed (Fig. 3f). We also did not find a correlation with levels of *Glycolysis* (Fig. 3g). Indeed, treating KCL fish with inhibitors PF-048599895 against the MAS, rotenone against complex I of the ETC or control DMSO between 3 dcpi and 7 dpci showed no difference in cardiomyocyte proliferation at 7 dpci (Fig. 3h). High levels of proliferation in the WIK were reflected in the largest reduction in wound length between 7 dcpi and 21 dpci (Fig. 3i). However, regeneration stalled after 21 dpci, and none of the hearts had fully regenerated by 90 dpci (Fig. 3j and Extended Data Figs. 1e and 4e). Therefore, cardiomyocyte proliferation plays a limited role during long-term regeneration, consistent with the knowledge that proliferation peaks at 7 dpci and substantially declines long before regeneration is completed^31^. These data suggest that increased OXPHOS is required for optimal cardiac regeneration after cessation of proliferation.

### Levels of OXPHOS correlate with the upregulation of border zone embryonic sarcomere expression

**Fig. 4:**
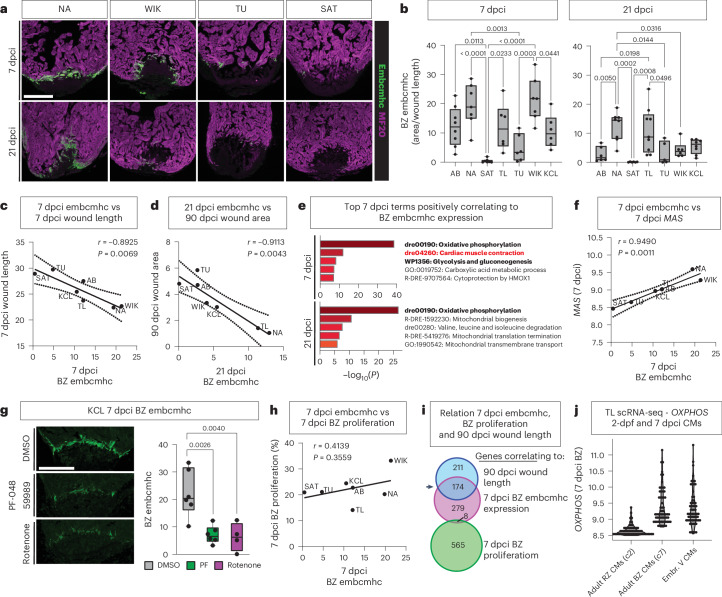
Levels of *OXPHOS* correlate to the upregulation of embryonic myosin. **a**, Representative images of MF20 and embcmhc double immunofluorescence staining showing differential embryonic myosin staining in the border zone cardiomyocytes of NA, WIK, TU and SAT at 7 dcpi and 21 dpci (scale bar, 300 µm). **b**, Quantification of differential expression of embcmhc in the border zone of the strains at 7 dpci and 21 dpci. **c**,**d**, Negative correlation between 7 dpci border zone embcmhc staining with 7 dpci wound length (**c**) and 21 dpci border zone embcmhc staining with 90 dpci wound area (**d**). **e**, Top five positively correlating terms in genes of 7 dpci bulk RNA-seq with 7 dcpi and 21 dpci border zone embcmhc activity. **f**, Positive correlation of embcmhc staining to *MAS* at 7 dpci. **g**, Reduced border zone embcmhc expression in 7 dpci KCL hearts treated with inhibitors PF-04859989 or rotenone compared to DMSO control. Representative images are on the left, and quantification is on the right (scale bar, 300 µm). **h**, No correlation between embcmhc activity and border zone cardiomyocyte proliferation at 7 dpci. **i**, Venn diagram displaying high overlap between genes correlating to 90 dpci wound length and 7 dpci embcmhc level but little overlap between embcmhc and border zone proliferation at 7 dpci. **j**, Levels of OXPHOS in adult 7 dpci remote and border zone cardiomyocytes and 2 dpf embryonic ventricular cardiomyocytes, showing similar levels of OXPHOS in border zone and embryonic cardiomyocytes. Reanalysis of previously published integrated scRNA-seq dataset^13^. **b**, 7 dpci: AB n = 8; NA, SAT, WIK *n* = 7; TL, TU, KCL *n* = 6, 21 dpci: AB, SAT, WIK *n* = 6; NA, KCL *n* = 8; TL *n* = 9; TU *n* = 5 (biological replicates); **g**, DMSO, PF-04859989 *n* = 6, rotenone *n* = 4 (biological replicates). **b**,**g**, One-way ANOVA with Tukey’s test. **c**,**d**,**f**,**h**, Simple linear regression. **e**, Analysis performed using Metascape. BZ, border zone; c, cluster; Embr. V. CM, embryonic ventricular cardiomyocyte; MF20, all cardiomyocytes; RZ, remote zone. Source data

As OXPHOS levels increase with cardiomyocyte differentiation^32^, and border zone cardiomyocytes de-differentiate and re-differentiate during regeneration^33,34^, we next analyzed the differentiation state of the border zone myocardium in which we identified *OXPHOS* activity. Embryonic cMHC antibody (embryonic cardiac myosin heavy chain (embcmhc), N2.261c) has been used to stain border zone cardiomyocytes, and its expression is considered to reflect their immature, embryonic state^18,35^. Staining with this antibody showed large differences among the strains at both 7 dcpi and 21 dpci (Fig. 4a,b). Especially notable was that, although expression in NA and WIK was similar at 7 dpci, it remained high at 21 dpci in NA but strongly declined in WIK, in which regeneration stalls at that stage (Fig. 4a,b and Extended Data Fig. 1e). By contrast, TU had low expression levels at 7 dpci, which further declined by 21 dpci, and expression was not evident in SAT at either stage (Fig. 4a,b). Border zone embcmhc expression at 7 dpci correlated with 7 dcpi and 90 dpci wound length, whereas 21 dpci embcmhc correlated with 90 dpci wound area (Fig. 4c,d). *OXPHOS* appeared to be driving embcmhc expression at 7 dcpi and 21 dpci (Fig. 4e and Supplementary Tables 6 and 7). *Glycolysis* and *MAS* gene expression positively correlated with 7 dpci embcmhc expression (Fig. 4e,f). Accordingly, there was significantly reduced embcmhc expression upon inhibition of the MAS (PF-04859989) or OXPHOS (rotenone) compared to DMSO control (Fig. 4g). Border zone embcmhc levels did not correlate to border zone proliferation (Fig. 4h). There was an overlap between genes correlating to 7 dpci embcmhc expression and 90 dpci wound length but not proliferation (Fig. 4i, arrowhead). These data reveal that higher and prolonged expression of embcmhc^18^ is beneficial for long-term regeneration.

**Extended Data Fig. 5:**
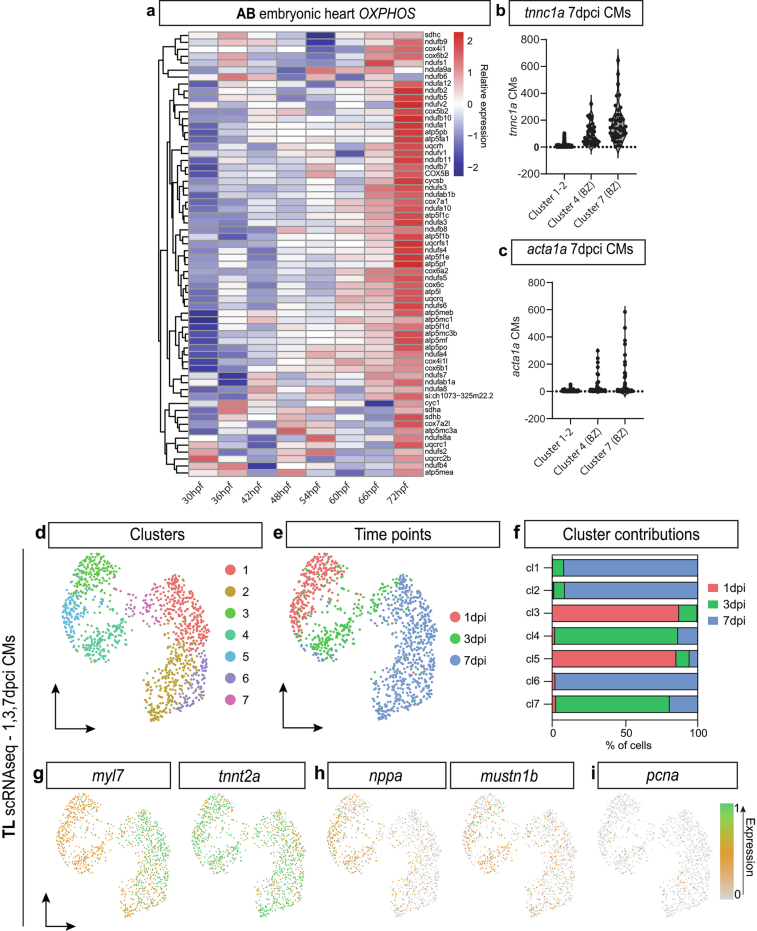
Cardiomyocyte proliferation is separated in time from OXPHOS and cardiomyocyte re-differentiation. (**a**) Heatmap of 7dpci border zone upregulated OXPHOS genes over time in the developing heart (re-analysed from Hill et al.).39 Re-analysed bulk RNAseq eight time points from 30 to 72hpf. (**b-c**) Violin plots showing the higher expression level of tnnc1a (**b**) and acta1a (**c**) in border zone (clusters 4 and 7) compared to remote zone cardiomyocytes (clusters 1 and 2) by re-analysis of our previous scRNAseq data. Cluster numbers as in original publication (Honkoop et al.). (**d**) UMAP plot showing the border zone cardiomyocytes grouped into seven clusters. (**e**) UMAP plot showing the distribution of the three time points. (**f**) Bar chart showing the temporal cluster contributions. (**g**) UMAP plot of cardiomyocyte specific genes myl7 and tnnt2a. (**h**) UMAP plot of border zone cardiomyocyte specific genes nppa and mustn1b. (**i**) UMAP plot of proliferation marker pcna. **d**-**i**, n=4 hearts per timepoint. Cl, cluster.

It seems counterintuitive that both MAS and OXPHOS appear to be required for this embryonic state, as embryonic cardiomyocytes have been shown to strongly rely on glycolysis instead of OXPHOS for their metabolism^36,37^. However, reanalysis of published bulk RNA-seq data of zebrafish heart development between 30 hours and 72 hours post-fertilization (hpf) from Hill et al.^38^ showed that *OXPHOS* genes are progressively upregulated during development, indicating that *OXPHOS* increases with cardiomyocyte maturation (Extended Data Fig. 5a). Furthermore, directly comparing 2 days post-fertilization (dpf) embryonic ventricular cardiomyocytes with 7 dpci border zone cardiomyocytes, using previously published integrated scRNA-seq data^13^, shows that *OXPHOS* levels in embryonic cardiomyocytes are similar to border zone cardiomyocytes and much higher than in remote zone cardiomyocytes (Fig. 4j). This indicates that *OXPHOS* levels indeed return to an embryonic state, but *OXPHOS* levels are unexpectedly high in cardiomyocytes during active (re-)differentiation. Upregulation of OXPHOS genes would facilitate the increase in proteins required for assembly of the ETC while mitochondrial numbers scale up to generate the energy required for sarcomere reassembly and maturation. The maintained high expression of embcmhc in strains such as NA and TL at 21 dpci indicates that a continued ‘embryonic’ re-differentiation state is beneficial for successful long-term regeneration, after proliferation levels have ceased.

### Temporal separation of cardiomyocyte proliferation, OXPHOS and re-differentiation

**Fig. 5:**
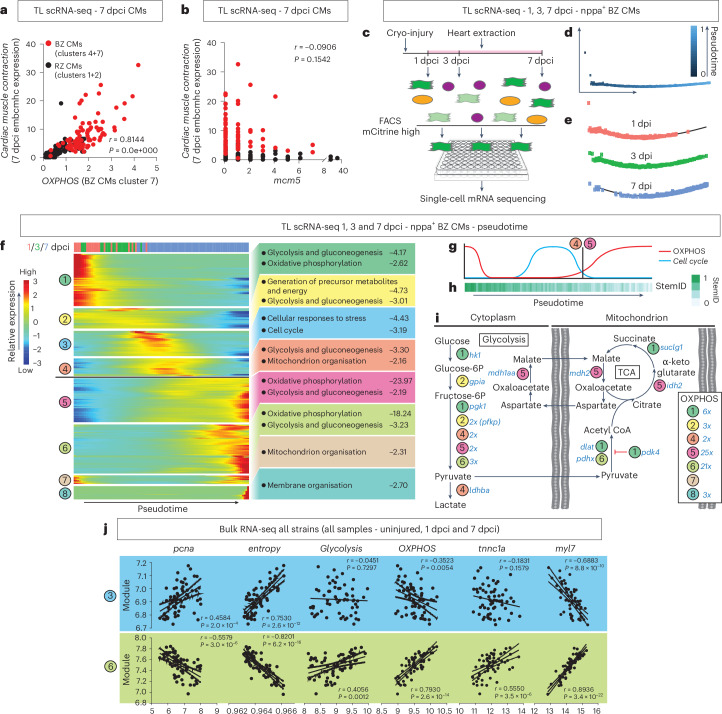
Cardiomyocyte proliferation is separated in time from OXPHOS and cardiomyocyte re-differentiation. **a**,**b**, Positive correlation of cardiac muscle contraction genes in cardiomyocytes to OXPHOS (**a**) but not to proliferation marker *mcm5* (**b**) by reanalyzing previous scRNA-seq data from Honkoop et al.^13^. Red indicates border zone cardiomyocytes; clusters are as in the original publication. **c**, Schematic diagram of the scRNA-seq procedure using the transgenic *TgBAC(nppa:mCitrine)* line to FACS 1 dcpi, 3 dcpi and 7 dpci border zone cardiomyocytes. **d**, Timeline generated by pseudotime analysis of the 1 dcpi, 3 dcpi and 7 dpci border zone cardiomyocytes. **e**, Pseudotime analysis recapitulates the real-time order of the samples. **f**, Differentially expressed genes changing over pseudotime, grouped into eight modules using unsupervised hierarchical clustering. Each module was analyzed for enriched processes. **g**, Schematic to indicate how levels of *OXPHOS* and *Cell cycle* genes alternate. **h**, StemID analysis shows that the cardiomyocytes differentiate along the pseudotime. **i**, Sequential activation of *Glycolysis*, *TCA cycle*, *MAS* and *OXPHOS* genes based on pseudotime analysis. **j**, Correlation graphs of strain bulk RNA-seq data comparing significantly expressed genes in modules 3 and 6 with proliferation (*pcna*), differentiation state (*entropy*), *Glycolysis*, *OXPHOS*, *tnnc1* (correlates to border zone embcmhc expression) and *myl7* (mature cardiomyocyte marker). Data showing positive correlation between proliferation and de-differentiation with module 3 but negative correlation to module 6. *OXPHOS* and *myl7* have the opposite pattern. Embryonic border zone marker *tnnc1a* only positively correlates to module 6 and, thus, the re-differentiation stage. **d**–**f**,**h**, *n* = 4 hearts per timepoint. **a**,**b**,**j**, Simple linear regression. BZ, border zone; CM, cardiomyocyte; RZ, remote zone.

Consistent with a correlation between long-term regeneration and cardiomyocyte re-differentiation, *Cardiac muscle contraction* as a Gene Ontology term was also enriched at 7 dpci (Fig. 4e, red). Although most of the genes in this term were *OXPHOS* genes, selecting only the genes with a specific role in muscle contraction showed that these were most strongly expressed in border zone cardiomyocytes and correlated to levels of *OXPHOS* (Fig. 5a and Extended Data Fig. 5b,c). By contrast, there was no correlation of expression of proliferation marker *mcm5* to levels of border zone *Cardiac muscle contraction* (Fig. 5b), indicating that proliferation and differentiation are mutually exclusive. To understand the timeline of events, we performed an scRNA-seq experiment using the *TgBAC(nppa:mCitrine)*^*8889Tg*^ line (TL background)^13^, in which mCitrine is expressed in border zone cardiomyocytes under the *nppa* promotor. mCitrine^+^ cardiomyocytes at 1, 3 and 7 dpci were FACS sorted and processed for scRNA-seq (Fig. 5c and Supplementary Information). Initial analysis identified seven clusters that appeared to be grouped according to the injury timepoints (Extended Data Fig. 5d–f and Supplementary Table 8). We confirmed that the cells were border zone cardiomyocytes and that a subcluster was entering the cell cycle (Extended Data Fig. 5g–i). To investigate the order of events that border zone cardiomyocytes undergo during regeneration, cells were projected into a linear pseudotime trajectory (Fig. 5d,e). We identified the genes that were differentially expressed over the pseudotime and grouped them with unsupervised hierarchical clustering into eight modules that were analyzed for enriched processes (Fig. 5f and Supplementary Tables 9–17). At 1 dpci, there was high expression of genes involved in *Glycolysis*, including *hk1*, encoding for a rate-limiting enzyme for glycolysis, and *pdk4*, encoding for an enzyme that inhibits pyruvate entry into the TCA cycle, likely due to cellular stress directly in response to injury (module 1)^39,40^. Between 1 dcpi and 3 dpci, *OXPHOS* genes were downregulated, whereas *Glycolysis* continued (module 2). During the transition from 3 dpci to 7 dpci, *Cell cycle* genes were upregulated (module 3). The peak of *Cell cycle* gene expression was directly followed by *Mitochondrial organisation* (module 4) and expression of large numbers of *OXPHOS* genes (module 5). *OXPHOS* as well as *Glycolysis* remained active toward the end of the 7-dpci timepoint (module 6). This was followed by further *Mitochondrial organisation* (module 7), possibly reflecting mitochondrial recovery^15^, and by *Membrane organisation*, suggesting cardiomyocyte remodeling, possibly to invade the wound (modules 8)^41–44^. These data indicate that when *OXPHOS* levels were lowest, *Cell cycle* was active (Fig. 5f,g). We then used the StemID algorithm, which revealed an increase in cardiomyocyte differentiation state along the pseudotime (lower diversity of cell fates and lower transcriptome entropy; Fig. 5h). Looking in detail at *Glycolysis*, *TCA* and *OXPHOS* genes in the pseudotime suggests sequential activation of the pathways (Fig. 5i). Although some *OXPHOS* and *TCA* genes were expressed in the initial injury response around the time of *Cell cycle* activity, the metabolic flow appeared to go toward lactate (module 4), followed by a sudden switch to genes of the MAS and TCA cycle in module 5 and a sudden strong upregulation of *OXPHOS* genes (Fig. 5f,g, black line). These data confirm that proliferation and OXPHOS are separated in time. Specific upregulation of *MAS* at the start of *OXPHOS* in the pseudotime supports our finding of a key role for the MAS in the activation of *OXPHOS* at 7 dpci.

**Extended Data Fig. 6:**
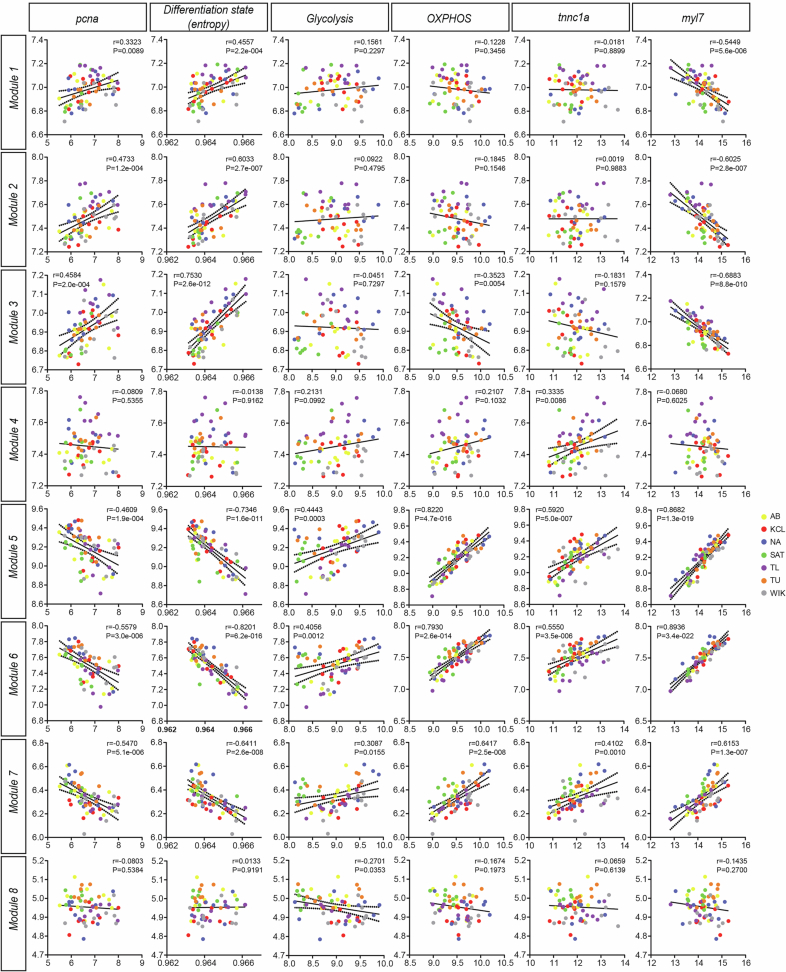
OXPHOS levels follow differentiation state of the cardiomyocytes. Bulk RNAseq data of the seven wild-type zebrafish strains, plotted against the eight pseudotime modules and the six processes of interest. All samples included (uninjured, 1- and 7dpci) and visible as separate dots coloured by strain. The significantly expressed genes per pseudotime module in the scRNAseq were identified in the bulk RNAseq, averaged per sample and plotted against the processes/genes of interest. This showed an increasing positive correlation of pcna and entropy in modules 1-3, before levelling off in module 4 and switching into a negative correlation in modules 5-8. The increasing positive correlation of entropy, which is a read out of de-differentiation, within modules 1-3 indicates that the cells increase their entropy/de-differentiate in these modules. The concurrent increasing positive correlation of pcna indicates that during this time, the cardiomyocytes initiate proliferation. Reversing of the correlations in modules 5-7 indicates that proliferation reduces and re-differentiation is initiated. There is no correlation of Glycolysis with modules 1-4, but a weak correlation with modules 5-7, indicating that Glycolysis is not associated specifically with the modules and is active throughout, albeit with a slightly stronger association to the later time points. OXPHOS and embryonic border zone marker tnnc1a showed an absent or weak negative correlation to modules 1-3, indicating these genes are not specifically associated with the de-differentiation and proliferation phase before switching into a positive correlation in modules 5-8. This indicates that OXPHOS and embryonic sarcomere border zone expression is specifically associated with the re-differentiation phase. Mature cardiomyocyte marker myl7 correlates strongly negatively to modules 1-3, confirming these modules represent the de-differentiation phase, before levelling off and switching into a strong positive correlation in modules 5-7 confirming this to be the re-differentiation phase. Simple linear regression.

Integrating the pseudotime data with the bulk RNA-seq of the different strains allowed us to plot the pseudotime modules against the processes of interest in the bulk RNA-seq (Fig. 5j and Extended Data Fig. 6). This revealed that genes from modules 1–3 positively correlated with both *pcna* and *entropy* levels (the higher the entropy, the more de-differentiated the cells) but negatively with levels of mature cardiomyocyte marker *myl7*, confirming that, during modules 1–3, cardiomyocytes de-differentiate and proliferate. There was a weak negative correlation to *OXPHOS* and no correlation to *Glycolysis*. In module 4, these correlations disappeared while reversing from module 5 onwards. Modules 5–7 showed a negative correlation to *pcna* and *entropy* and a positive correlation to *myl7*, indicating cardiomyocyte re-differentiation and cell cycle exit. There was a strong correlation between levels of *Glycolysis* and *OXPHOS* with these later modules. The marker of embcmhc expression, *tnnc1a*, did not correlate to the early pseudotime or to module 3, when the cells are most de-differentiated, indicating that it is not a de-differentiation marker. By contrast, it correlated to modules 5–7, confirming that upregulation of embryonic sarcomere genes and *OXPHOS* are specific for cardiomyocyte re-differentiation.

### Genetic manipulation of *mdh1ab* confirms its role in long-term regeneration

**Fig. 6:**
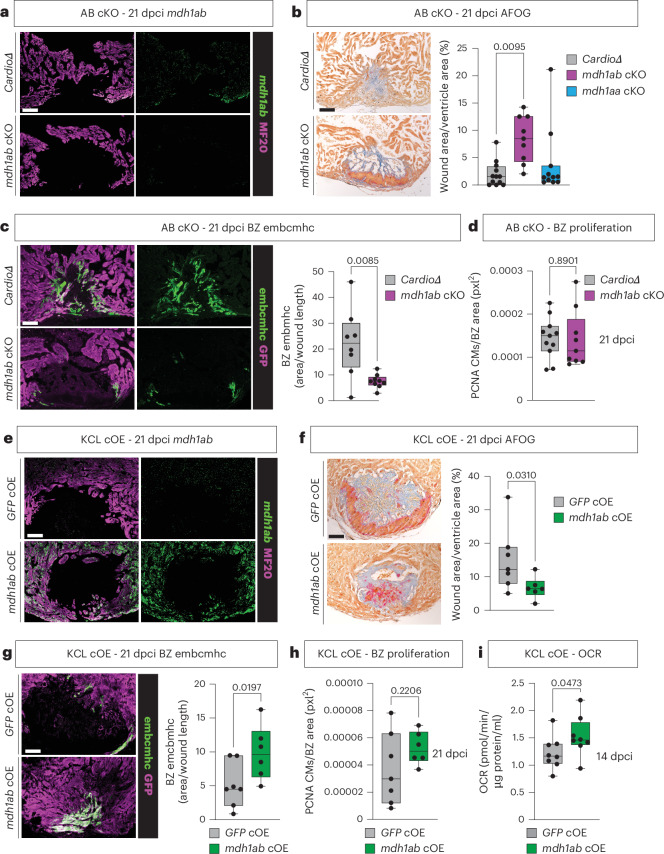
Genetic manipulation of *mdh1ab* confirms its role during long-term regeneration and cardiomyocyte re-differentiation. **a**, Representative images of RNAscope analysis of AB cardiomyocyte-specific *mdh1ab* knockout and *cardiodeleter* control showing absence of *mdh1ab* mRNA in the MF20^+^ myocardium in the knockout. **b**, Representative images of AFOG staining on AB cardiomyocyte-specific *mdh1ab* knockout and *cardiodeleter* control with wound area quantification showing increased 21 dpci wound area in the *mdh1ab* knockout but not the *mdh1aa* knockout in comparison to controls. **c**, Representative images of embchmc immunohistochemistry on AB cardiomyocyte-specific *mdh1ab* knockout and *cardiodeleter* control, with quantification showing reduced presence of embcmhc in the knockout compared to control. **d**, Quantification of PCNA^+^ border zone cardiomyocytes indicates no differences in proliferation between the AB cardiomyocyte-specific *mdh1ab* knockout and the *cardiodeleter* control. **e**, Representative images of RNAscope analysis of KCL cardiomyocyte-specific *mdh1ab* overexpression and GFP control showing increased levels of *mdh1ab* mRNA in the MF20^+^ myocardium in the overexpression heart. **f**, Representative images of AFOG staining on KCL cardiomyocyte-specific *mdh1ab* overexpression and GFP control, with wound area quantification showing reduced 21 dpci wound area in the *mdh1ab* overexpression model compared to controls. **g**, Representative images of embchmc immunohistochemistry on KCL cardiomyocyte-specific *mdh1ab* overexpression and GFP control, with quantification showing reduced presence of embcmhc in the overexpression hearts compared to control. **h**, Quantification of PCNA^+^ border zone cardiomyocytes indicate no differences in proliferation between the KCL cardiomyocyte-specific *mdh1ab* overexpression and GFP control. **i**, OCR measurements of 14-dpci ventricles show increased levels of OXPHOS in the overexpression hearts compared to control (scale bars, 100 μm). **b**, *mdh1ab* cKO *n* = 9, *mdh1aa* cKO *n* = 11, *cardiodeleter* *n* = 12 (biological replicares); **c**, *mdh1ab* cKO *n* = 9, *cardiodeleter* *n* = 8 (biological replicates); **d**, *mdh1ab* cKO *n* = 9, *cardiodeleter* *n* = 11 (biological replicates); **f**,**h**, *mdh1ab* cOE *n* = 6, *GFP* cOE *n* = 7 (biological replicates); **i**, *n* = 8 biological replicates per group. **b**, One-way ANOVA with Tukey’s test. **c**,**f**,**i**, Unpaired one-tailed Welchʼs *t*-test. **d**,**h**, Unpaired two-tailed Student’s *t*-test. **g**,**i**, Unpaired one-tailed Student’s *t*-test. BZ, border zone; CM, cardiomyocyte. Source data

**Extended Data Fig. 7:**
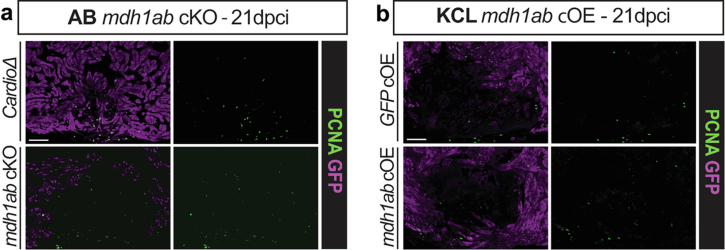
Genetic manipulation of mdh1ab has no effect on cardiomyocyte proliferation. (**a**) Representative images of immunofluorescence staining of mdh1ab cKO and cardiodeleter 21dpci hearts labelling PCNA-positive proliferating cells and GFP-positive myocardium. (**b**) Representative images of immunofluorescence staining of mdh1ab cOE and GFP cOE 21dpci hearts labelling PCNA-positive proliferating cells and GFP-positive myocardium. Scalebars: 100μm.

Our data indicate that activation of the MAS and OXPHOS are key for re-differentiation and long-term regeneration. However, as both inhibitors might have off-target effects^30,45,46^, we generated cardiomyocyte-specific knockouts (cKOs) for *mdh1ab* (*mdh1ab* cKO, AB strain) (Fig. 6a). To generate the knockout, we injected a construct containing three gRNAs to remove the entire *mdh1ab* gene into a *cardiodeleter* line expressing Cas9 under control of the *myl7* promoter^47^. Quantification of wound size at 21 dpci showed reduced regeneration in the *mdh1ab* cKO, but not the *mdh1aa* cKO, compared to control *cardiodeleters*, confirming our findings using the MAS inhibitor (Fig. 6b). Further quantification of the hearts for embcmhc showed a strong reduction in embcmhc staining in the border zone cardiomyocytes in absence of *mdh1ab* (Fig. 6c), whereas cardiomyocyte border zone proliferation was unaffected (Fig. 6d and Extended Data Fig. 7a). To understand if manipulating *mdh1ab* could result in enhanced regeneration, we also generated a cardiomyocyte-specific *mdh1ab*-overexpressing line (*mdh1ab*cOE, KCL strain) (Fig. 6e). Overexpression in cardiomyocytes indeed resulted in enhanced regeneration compared to green fluorescent protein (GFP)-overexpressing controls (Fig. 6f). Correspondingly, embcmhc was increased, whereas proliferation was again not different (Fig. 6g,h and Extended Data Fig. 7b). To confirm the role of *mdh1ab* in the upregulation of OXPHOS, we measured the OCRs of the overexpression and control hearts at 14 dpci, which indeed showed increased OXPHOS rates (Fig. 6i). Together, these data validate the role of the MAS in the upregulation of OXPHOS, cardiomyocyte differentiation and successful regeneration.

### Reduced upregulation of *OXPHOS* and initiation of border zone re-differentiation in cavefish

**Fig. 7:**
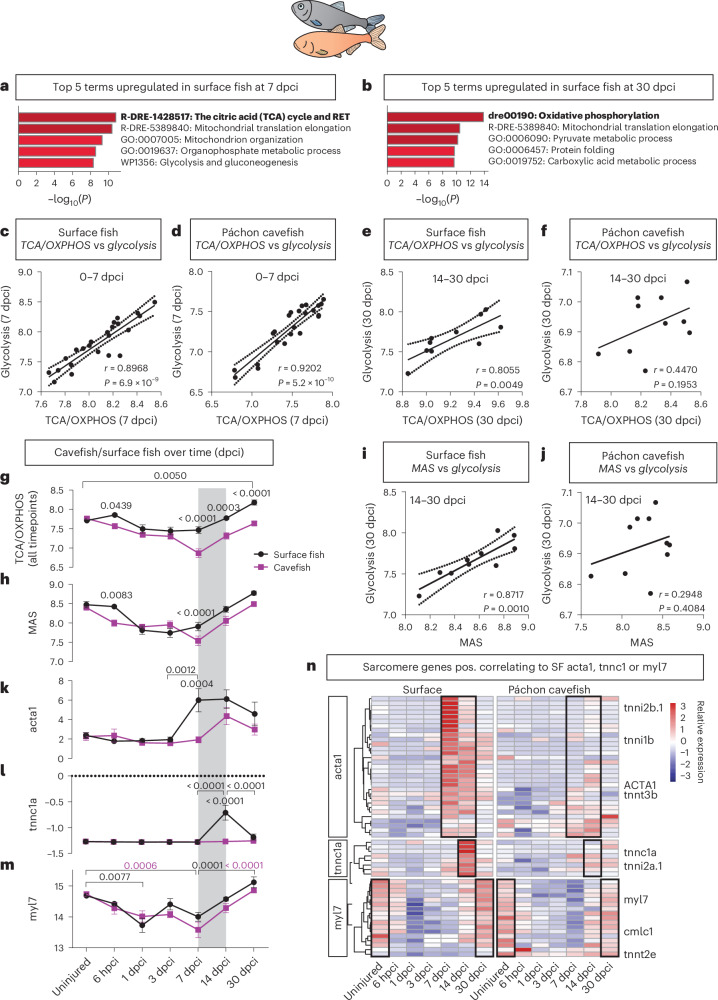
Strongly reduced re-differentiation gene expression in *A. mexicanus* cavefish versus surface fish. **a**,**b**, Top five enriched processes upregulated in surface fish at 7 dpci (**a**) and 30 dpci (**b**) compared to cavefish. **c**–**f**, Positive correlation of TCA/OXPHOS with *Glycolysis* genes at all timepoints in surface fish (**c** and **e**) but only up to 7 dpci in cavefish (**d** and **f**). **g**,**h**, Expression over time in bulk RNA-seq data. Although TCA/OXPHOS genes are initially relatively similarly expressed, they dip in cavefish at 7 dpci. From 7 dpci onwards, there is a steady increase until at least 30 dpci (**g**). Expression of MAS genes follows a similar pattern as TCA/OXPHOS expression (**h**). **i**,**j**, Significant positive correlation between the *MAS* and *Glycolysis* at late timepoints in surface fish (**h**) but not in cavefish (**i**). **k**–**m**, Expression over time in bulk RNA-seq data of two genes correlating to embcmhc expression in zebrafish, *acta1* (**k**) and *tnnc1a* (**l**), showing strong upregulation at late timepoints in surface fish but much lower and shorter upregulation in cavefish. Mature marker *myl7* expression reduces in the first days after injury, before increasing again from 7 dpci onwards (**m**). Selected significance is shown. **n**, Heatmap of sarcomere and myosin-related genes correlating to *acta1*, *tnnc1a* or *myl7* in surface fish. Timepoints of interest are highlighted with black box. **a**,**b**, *n* = 5 biological replicates per morph per timepoint; **g**,**h**,**k**–**m**, *n* = 3 for uninjured cavefish and surface fish, *n* = 5 per morph for all other timepoints (biological replicates). **a**,**b**, Analysis performed using Metascape. **c**–**f**,**i**,**j**, Simple linear regression. **g**,**h**,**k**–**m**, Two-way ANOVA with Sidakʼs multiple test; data presented as mean ± s.e.m. pos., positively. SF, surface fish.

**Extended Data Fig. 8:**
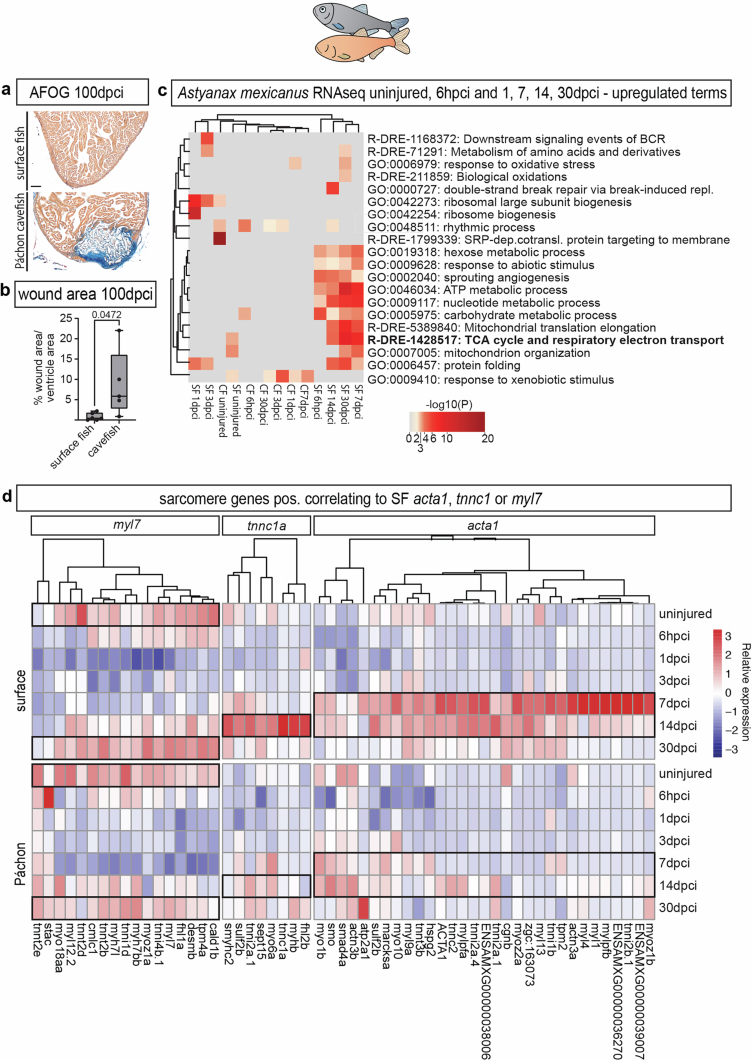
Strongly reduced re-differentiation gene expression in Astyanax mexicanus cavefish versus surface fish. (**a**) Representative AFOG images displaying the scarring wound in Astyanax mexicanus Pachon cavefish compared to the regenerating surface fish heart at 100dpci (scale bar: 100μm). (**b**) Quantification of wound area, showing significant difference in wound size between surface fish and Pachon cavefish. (**c**) Heatmap comparing all time points in both cavefish and surface fish showing upregulated terms including TCA cycle and respiratory electron transport among the top terms in surface fish at late time point after cryoinjury. (**d**) Same as Fig. 7n, but with all genes annotated in heatmap. **b**, Surface fish n=6; Cavefish n=5, (biological replicates); **b** unpaired one-tailed Welch t-test; **c**, analysis performed using Metascape. Source data

To confirm our findings and to investigate potential evolutionary conservation of a beneficial role of OXPHOS during heart regeneration, we used *A. mexicanus*. We confirmed our previous findings that the surface fish population of *A. mexicanus* can regenerate its heart after ventricular resection, whereas the Páchon cavefish population forms a permanent scar^48^ using cryo-injury (Extended Data Fig. 8a,b). Next, we performed bulk RNA-seq on *A. mexicanus* hearts from 6 hours to 30 dpci. Analysis of the top enriched processes revealed the terms *The citric acid cycle and respiratory electron transport* and *Oxidative phosphorylation* (further referred to as *TCA/OXPHOS*) as the most strongly upregulated processes at late timepoints in the regenerative surface fish compared to cavefish (7, 14 and 30 dpci; Fig. 7a,b, Extended Data Fig. 8c and Supplementary Tables 18–20). In the first 7 days after injury, levels of *TCA/OXPHOS* correlated to the levels of *Glycolysis* in both surface fish and cavefish (Fig. 7c,d); however, this was lost in cavefish at later timepoints (Fig. 7e,f), similar to the observation in the SAT zebrafish strain.

There was a steady increase in TCA/OXPHOS from 7 dpci onwards in the regenerating surface fish, elevating above baseline and still at an upward trend at 30 dpci (Fig. 7g). In the scarring cavefish, levels of *TCA/OXPHOS* were similar to surface fish in the first 3 days; however, at 7 dpci, there was a sudden decline followed by an increase that was lower than the surface fish (Fig. 7g). *MAS* showed a very similar pattern to *OXPHOS* (Fig. 7h), and, whereas *MAS* correlated to *Glycolysis* at late timepoints in surface fish, this correlation was lost in cavefish (Fig. 7i,j), suggesting that glycolysis does not directly fuel into OXPHOS via the MAS anymore.

To understand if this could affect cardiomyocyte re-differentiation, we plotted sarcomere markers *acta1* and *tnnc1a* that correlated to 7 dpci embcmhc expression in zebrafish over time in *Astyanax* (Fig. 7k,l)^44^. In surface fish, *acta1* was upregulated at 7 dpci and started to decline after 14 dpci, whereas, in cavefish, the upregulation was lower, delayed and shorter (Fig. 7k). *Tnnc1a* was upregulated later and shorter than *acta1* in surface fish, at 14 dpci only, but completely absent in cavefish (Fig. 7l). This indicates that at the time when the correlation between *Glycolysis* and *OXPHOS* is lost, non-regenerative cavefish show a dampened upregulation of temporary ‘embryonic’ sarcomere genes, similar to the zebrafish strains SAT and TU (least regenerative). Plotting mature sarcomere marker *myl7* expression confirmed the decline in the first days after injury when the cardiomyocytes are de-differentiating, before increasing steadily again from 7 dpci onwards, and with an upward trend at 30 dpci, signaling the maturation of sarcomeres during re-differentiation (Fig. 7m). We then looked for sarcomere genes correlating to *acta1*, *tnnc1a* or *myl7* in surface fish (*r* > 0.7), which showed that there was a dynamic temporary sarcomere gene program correlating to *acta1* and *tnnc1a* that was strongly reduced in cavefish (Fig. 7n and Extended Data Fig. 8d). Levels of ‘mature’ sarcomere genes correlating to *myl7* were upregulated in the later stages in cavefish but did not achieve the strong increase between 14 dcpi and 30 dpci that was seen in surface fish (Fig. 7n). This shows that, during the re-differentiation phase in regenerative fish, a dynamic temporal sarcomere gene program is activated, which is dampened when *OXPHOS* levels fail to increase sufficiently.

## Discussion

Inter-species comparisons are a powerful approach to discover novel mechanisms involved in regeneration. However, it is difficult to tease out causative pathways when comparing highly diverse species such as zebrafish and mouse. These difficulties disappear when performing intra-species comparisons^49^. We previously showed the power of comparing within a species using *A. mexicanus* surface fish and cavefish, which led to the discovery of a conserved role for *lrrc10* during cardiomyocyte re-differentiation and regeneration^34,48^. In the present study, we took this approach further by comparing seven different wild-type zebrafish strains. Although zebrafish is considered the model organism for adult heart regeneration research^50^, we show that not all zebrafish regenerate equally. Taking advantage of the regenerative range observed in the different zebrafish strains and *A. mexicanus* has allowed us to understand seemingly contradicting processes of successful cardiomyocyte proliferation and upregulation of *OXPHOS* during heart regeneration.

By integrating single-cell and bulk RNA-seq zebrafish data to generate a timeline of events, we identify a downregulation of *OXPHOS* during de-differentiation of the border zone cardiomyocytes and confirm the maintenance of *Glycolysis* during proliferation^13,14^. Cardiomyocyte proliferation peaks when *OXPHOS* levels are lowest, followed by a sudden increase in *OXPHOS* toward the end of day 7. As ROS generated by OXPHOS inhibit cardiomyocyte proliferation^7^, the upregulation of OXPHOS could function to prevent hyperplasia.^14^ After the peak at 7 dpci, cardiomyocyte proliferation levels return to baseline by 21–30 dpci^31^. This is consistent with our finding that cardiomyocyte proliferation is important for regeneration until 21 dpci, but its role is negligible at later stages. Although this window of proliferation appears relatively short, it is sufficient to restore cardiomyocyte numbers to pre-injury levels by 30 dpci^31^. Interestingly, high levels of proliferation do not necessarily correlate with successful long-term regeneration, as exemplified by the WIK strain, which, although highly proliferative, does not complete regeneration. Reducing OXPHOS through knockout of *cox7a1* increases cardiomyocyte proliferation and improves regeneration at 28 dpci^51^. However, long-term regeneration was not analyzed, and it will be interesting to investigate the ability of cardiomyocytes to re-differentiate in these knockouts.

In contrast to proliferation, expression of embcmhc correlates well to long-term regenerative outcome. Embcmhc expression has been considered a hallmark of de-differentiation of the border zone cardiomyocytes, as they revert to a more embryonic state^18,35^. As embryonic cardiomyocytes are thought to largely rely on glycolysis, with low OXPHOS levels^15,37^, the upregulation of *OXPHOS* in the border zone and correlation to embcmhc expression seemed counterintuitive. However, we found that embryonic *OXPHOS* levels are higher than those of adult cardiomyocytes away from the injury site and similar to those of the border zone, indicating that *OXPHOS* levels are higher in actively (re-)differentiating cells, which are reassembling their sarcomeres, as compared to fully differentiated cardiomyocytes. Thus, border zone cardiomyocytes do indeed revert to an embryonic state, but, in contrast to current beliefs, this results in an upregulation of *OXPHOS* that is important for long-term regeneration.

Sarcomere gene isoforms are known to switch as development and cardiomyocyte maturation progresses, with embryonic isoforms downregulated in the adult heart and replaced by expression of mature isoforms^34,52^. Our data in *Astyanax* show that this switch also occurs during re-differentiation, with high, but temporal, upregulation of many sarcomere isoforms between 7 dpci and 30 dpci, whereas levels of other mature isoforms slowly increase until they become the main isoforms at 30 dpci. Temporal ‘embryonic’ sarcomere gene expression is not only higher in regenerative surface fish versus non-regenerative cavefish but also longer in duration. Similarly, in zebrafish, high and prolonged expression of embcmhc is beneficial to regeneration and especially long-term outcome. The transient embryonic sarcomere profile could be required for functional cardiomyocyte assembly and reintegration within the three-dimensional continuously contracting cardiac muscle. Studies on isoform switching of sarcomeres in mice suggest that embryonic/fetal isoforms may be less stiff and rigid, which could promote cardiomyocyte migration and functional reintegration during cardiac repair^52^.

High levels of OXPHOS allow the border zone cardiomyocytes to complete a full differentiation process that mimics developmental maturation. If OXPHOS levels remain too low, full differentiation is not achieved, which fits well with the finding from zebrafish that, in contrast to those completely regenerated, incompletely regenerated hearts still contain immature cardiomyocytes near the wound at 90 dpci^31^. In contrast to zebrafish, non-regenerative mouse and human wound border zone cardiomyocytes fail to fully re-differentiate^34^. Similarly, mouse cardiomyocytes that have de-differentiated due to transient exposure to constitutively active ERBB2 fail to fully re-differentiate^53^. The lack of re-differentiation in adult mouse, but not zebrafish, cardiomyocytes seems ironic, knowing that the mouse heart relies heavily on use of OXPHOS for its energy^54^.

Our data point to a central role for the MAS in the sudden upregulation of OXPHOS. The MAS, which indirectly transfers NADH into the mitochondria and regenerates NAD^+^ in the cytoplasm^29,55^, ensures maximal ATP generation from carbohydrate substrates by coupling with the ETC, thereby capitalizing on the capacity for glycolytic metabolism in fish. Although our study focuses on the boost in OXPHOS generated by activation of the MAS, the increase in TCA metabolites will likely play a wider role, from mediating biosynthetic pathways to regulation of chromatin modifications that can impact cell state and function, which requires further investigation. With a much more limited ability to upregulate glycolysis^54^, adult mouse cardiomyocytes can likely not fully benefit from activation of the MAS to obtain the levels of OXPHOS required for full sarcomere assembly. Our data show that cardiomyocytes undergo different phases during regeneration, with dynamic metabolic rewiring between the early and late stages. During the early stages, upregulation of glycolysis and downregulation of OXPHOS is key for successful cardiomyocyte proliferation. However, long-term completion of regeneration requires increased rates of OXPHOS during sarcomere reassembly and restoration of cardiac function, before returning to the pre-injury adult metabolic state. As OXPHOS has been considered detrimental to regeneration, our findings require a shift in thinking about approaches in targeting pathways to induce long-term heart repair in the human heart.

## Methods

### Animals

Zebrafish and *A. mexicanus* were used in this study. Six wild-type zebrafish strains—AB, NA, SAT, TL, TU and WIK—were obtained as embryos from the Zebrafish International Resource Center and from John Postlethwait’s laboratory (University of Oregon). KCL has been bred in our facility for more than 20 generations but originated from King’s College London. The wild-type zebrafish and transgenic *TgBAC(nppa:mCitrine)*^*8889Tg*^ (ref. ^13^) and *Tg(myl7:NLS-GFP-2A-NLS-Cas9-NLS)*^*bcz101Tg*^ (*cardiodeleter*)^47^ zebrafish lines were kept at 28 °C under a 10/14-hour light/dark cycle according to current guidelines^56^. *A. mexicanus* surface and cavefish were maintained at 20–21 °C under a light/dark cycle of 12/12 hours. All procedures involving animals at the University of Oxford were carried out in compliance with the revised Animals (Scientific Procedures) Act 1986 in the United Kingdom and Directive 2010/63/EU in Europe and were approved by Oxford University’s central Committee on Animal Care and Ethical Review. All procedures performed in the Hubrecht Institute were approved by the Animal Welfare Body of the Royal Dutch Academy of Sciences and Arts in compliance with animal welfare laws, guidelines and policies, according to Dutch and European law. Ethical approval was also obtained from the Institutional Animal Care and Use Committees of Massachusetts General Hospital and Boston College.

### Generation of transgenic zebrafish strains

To generate cardiomyocyte-specific mutants, guide shuttles carrying three gRNAs targeting either *mdh1aa* or *mdh1ab* were assembled and injected as previously described^47^. In short, three gene-specific gRNAs without predicted off-targets in the zebrafish genome were selected using CRISPRscan^57^ and subsequently cloned in vectors containing the *U6a*, *U6b* and *U6c* zebrafish promoters. These U6–gRNA cassettes were then subcloned and combined using Gibson Assembly into a Tol1-based construct carrying a transgenesis reporter that labels cardiomyocytes with the red fluorescent protein mKate. The resulting *pTol1-U6abc-mdh1aa-cmlc2:n-mKate* and *pTol1-U6abc-mdh1ab-cmlc2:n-mKate* plasmids were injected into *cardiodeleter* embryos at the one-cell stage with *Tol1* mRNA. Injected animals were screened at 72 hpf to select embryos showing mKate expression in cardiomyocytes, raised to adulthood and screened for transmission. F_1_ animals carrying the *cardiodeleter* and corresponding guide shuttle were used for regeneration experiments. Targeted regions (including PAM) are as follows: GTTTTGGTGACTGGCGCCGCCGG, GGGCATAGAGCCCACCAAGATGG and TGTTGTGGTCCAGACGGGTCAGG for *mdh1aa* and TGCGATCTGTCCTGCGGCACCGG, TGGCTGGGTTGCCAACAACTAGG and GTGAAGAATGTGATCATCTGGGG for *mdh1ab*.

To generate cardiomyocyte-specific overexpression models, *pTol2-cmlc2-mdh1ab-P2A-GFP-Tol2* and *pTol2-cmlc2-GFP-Tol2* were designed and ordered from VectorBuilder, transformed as previously described^58^. *Escherichia coli* HST08 Stellar Competent cells (TaKaRa) were incubated for 30 minutess with 5 ng of the plasmid at 4 °C for 30 minutes. The cells were heat shocked at 42 °C for 45 seconds, cooled down for 2 minutes at 4 °C, incubated for 1 hour at 37 °C in SOC medium (TaKaRa) and plated on LB agar plates (Q-BIO gene) containing 80 μg ml^−1^ carbenicillin (Thermo Fisher Scientific) overnight at 37 °C. Single colonies were expanded in LB broth overnight at 37 °C, and DNA was isolated using a QIAprep Spin Miniprep Kit (Qiagen) according to the manufacturer’s instructions. pCS2-Tol1 and pCS-Tp-Tol2 plasmids were linearized with NotI-HF (New England Biolabs) and purified using a QIAquick PCR Purification Kit (Qiagen) according to the manufacturer’s instructions. In vitro transcription of the Tol1/Tol2 genes was achieved using an mMESSAGE mMACHINE SP6 Transcription Kit (Invitrogen). DNA and mRNA concentration was assessed using a NanoDrop 2000 (Thermo Fisher Scientific). Microinjection of zebrafish embryos was performed as previously described^59^. Single-cell-stage zebrafish embryos were injected (FemptoJet 4x; Eppendorf) with a mix of 50 ng μl^−1^ plasmid DNA and 50 ng μl^−1^ Tol2 RNA. Founder embryos were bred to wild-type zebrafish to produce the F_1_ generation stably expressing the transgene(s).

### Cryo-injury of zebrafish and *A. mexicanus* heart

Zebrafish (5 months–1.5 years old) and *A. mexicanus* (2 years old) were used, age matched within experiments. Cryo-injury of the ventricle of zebrafish and *A. mexicanus* heart was performed per previous description^60^. In brief, fish were anesthetized in MS-222 (250 mg l^−1^; Sigma-Aldrich) and placed ventral side facing upwards in a sponge-holder under a dissection microscope (Olympus). An incision was made using forceps and microdissection scissors at the level of the heart, which facilitated the exposure of the ventricle out of the pericardial cavity. Cryo-injury was induced on the tip of the ventricle using a copper probe pre-chilled in liquid nitrogen. In sham groups, a probe at room temperature was used. After surgery, fish were returned to fresh tank water immediately, and their gills were pipetted through using water to facilitate breathing and restoring of swimming capability. Hearts were isolated at the indicated timepoints and processed for further analysis.

### Heart sample fixation, processing and histology sections

Isolated hearts were rinsed in pre-cooled PBS and fixed in 4% paraformaldehyde (ChemCruz) overnight at 4 °C. Dehydration was performed using increasing ethanol concentrations (70%, 80%, 90%, 96% and 100%). After overnight incubation in 100% 1-butanol, the samples were placed in paraffin (Paraplast; Sigma-Aldrich) at 65 °C, and 8-μm-thick sections were acquired using a Microm HM325 microtome. The sections were mounted on SuperFrost glass slides (VWR) and dried overnight at 37 °C for further processing.

### Acid Fuchsin Orange G staining

After sectioning the whole heart, exactly one in 10 sections was mounted per set, providing representative coverage of the whole heart for Acid Fuchsin Orange G (AFOG) staining. The procedure was performed as previously described^60^. In brief, the samples were de-waxed twice for 6 minutes in Histo-Clear II (National Diagnostics), rehydrated in descending concentration of ethanol (100%, 96%, 90%, 80% and 70%, 1 minute each), fixed in Bouin’s solution (Sigma-Aldrich) at 60 °C for 2 hours and stained in AFOG solution (0.5% w/v methyl blue, 1% w/v orange G and 1.5% w/v acid fuchsin (pH 1.09)) for 7 minutes. The stained slides were dehydrated in ascending concentration of ethanol (70%, 80%, 90%, 96% and 100%, 1 minute each) and washed twice for 6 minutes in Histo-Clear II. Finally, the slides were briefly allowed to dry before being mounted in dibutylphthalate polystyrene xylene (DPX) medium (Sigma-Aldrich) for imaging.

### Immunofluorescence staining

For immunofluorescence analysis, we took at least three sections per heart with typical wound for further processing. The samples were de-waxed with xylene (Sigma-Aldrich) and rehydrated as described above. The sections were boiled in antigen unmasking solution, citric acid based (Vector Laboratories), for 4 minutes in a pressure cooker, followed by a cool-down period in PBS for 20 minutes. The sections were then incubated in TNB (Akoya Biosciences) blocking buffer (0.1 M Tris base (pH 7.4), 0.15 M NaCl, 0.5% TNB) for 30 minutes to 1 hour at room temperature to prevent non-specific binding. Incubation with primary antibody solution was performed in a humidified chamber at room temperature overnight. After washing with PBST (containing 0.1% Tween 20), sections were incubated with secondary antibodies for 2 hours at room temperature. Primary antibodies Mef2c (Biorbyt, orb256682), PCNA (clone PC10; Dako, M0879), GFP (Abcam, ab13970), MF20 (Developmental Studies Hybridoma Bank (DSHB), AB_2147781) and embcmhc N2.261 (DSHB, AB_531790) and secondary antibodies Alexa Fluor 488 (Invitrogen, anti-mouse A11001, anti-chick A11039 and anti-rabbit A21206) and Alexa Fluor 555 (Invitrogen, A31570) were prepared using TNB buffer at a ratio of 1:200. When the staining was ready, the sections were stained using DAPI (1:1,500 in TNB) for 5 minutes and were mounted in a self-made mounting medium, Mowiol 4-88 (AppliChem), for imaging.

### RNAscope

For RNAscope, we mounted three sections per heart with typical wound for further processing. The samples were deparaffinized in xylene, incubated in 100% ethanol and air dried. After a 10-minute incubation in H_2_O_2_, the samples were boiled in target retrieval (Bio-Techne) for 15 minutes, washed in ethanol and allowed to dry. The *mdh1ab* (Bio-Techne, 1175851-C1) zebrafish probe was applied at 40 °C (HybEZ II oven; ACD) after a 12-minute incubation with protease III (Bio-Techne). Amp1, Amp2 and Amp3 were added before incubating in HRP-C1, TSA-fluorescein and HRP blocker at 40 °C (Bio-Techne). Two washes in RNAscope wash buffer (Bio-Techne) were performed in between each step. Immunofluorescence staining was then performed, and the slides were mounted with ProLong Gold antifade mounting medium (Invitrogen).

### Drug treatment

The KCL strain was used for all inhibitor experiments. The drug solutions of PF-04859989 (Sigma-Aldrich, 0.2 µM) and rotenone (MP Biomedicals, 5 µM) were prepared in 0.01 µM DMSO (Sigma-Aldrich) in Milli-Q water. The drugs were then injected intraperitoneally, together with a control DMSO (0.01 µM) group using a BD Micro-Fine U-100 insulin syringe (29-gauge) with injection volume of 10 μl. For the 7-dpci heart isolation, fish were injected once daily from 3 dpci to 7 dpci. For 21-dpci isolation, hearts were injected every other day between 1 dpci and 20 dpci.

### Bulk RNA-seq and data analysis

Both zebrafish and *A. mexicanus* hearts were harvested at desired timepoints, and total RNA was extracted from individual ventricles using a Quick-RNA MicroPrep Kit (Zymo Research). The quality of RNA was assessed using a NanoDrop 2000 (Thermo Fisher Scientific), a Qubit RNA HS assay (Thermo Fisher Scientific) and an Agilent 2100 Bioanalyzer. We used 1.5 μg of total RNA. Libraries were generated using a QuantSeq 3′ mRNA-Seq Library Prep Kit (FWD) from Lexogen. The zebrafish libraries were then sequenced using two lanes on the NovaSeq 6000 platform and those of *A. mexicanus* on the Illumina HiSeq 4000 by Oxford Genomics at the Wellcome Centre for Human Genetics. Reads were demultiplexed using bcl2fastq (version 2.20.0, Illumina) and checked for quality (adapter trimming and quality control with Trim Galore (version 0.6.4_dev))^61^ before alignment and gene counting using STAR (version 2.7.6a)^62^ against the zebrafish genome (GRCz11) with Ensembl (https://ensembl.org) annotations (release 104). For *Astyanax*, alignment was against the Mexican tetra (version 2.0) genome with customized Ensembl annotations (release 103; see GSE234989 for table)^63^. Raw reads and gene counts are accessible under accession number GSE234990 in the Gene Expression Omnibus (GEO) repository. Gene counts were loaded in R (version 4.0.1)^64^, and genes with less than one read per million mapped in all samples were removed before further analysis. Differential gene expression (DGE) was determined using DESeq2 (ref. ^65^) after controlling for unwanted variation with RUVg^66^, using genes with a coefficient of variation less than the median as negative controls for RUVg. The weight matrix generated by RUVg was added to the linear model for DESeq2 DGE analysis. Genes were deemed to be differentially expressed if they showed more than two-fold differences at a Benjamini–Hochberg^67^ adjusted *P* < 0.05. Gene Ontology enrichment was evaluated using Metascape (http://metascape.org) using zebrafish annotations and a background comprising genes retained for analysis^26^. For KEGG pathway analysis^68–70^, differential expression analysis was performed using the generalized linear models in edgeR (version 4.3.1)^71^. Genes with fold change > 1.5 and false discovery rate < 0.1 were extracted for enrichment analysis. The enrichment analysis was performed using EnrichR version 3.4.0 (ref. ^72^) on the zebrafish KEGG 2019 dataset, with default parameters. Genome-scale metabolism modeling (GEM) was performed using the continuous_integration scoring strategy in troppo version 0.0.7 (BioSystemsUM; Tissue-specific RecOnstruction and Phenotype Prediction using Omics data; https://github.com/BioSystemsUM/troppo; accessed 27 February 2025) on the ZebrafishGEM model^73^, with the raw RNA-seq counts as input. Heatmaps were produced using pheatmap^74^ in R. Venn diagrams were made with R packages Vennerable (proportional Venn^75^) or gplots (petal plots^76^). For plots of expression pattern correlation with *acta1*, *tnnc1a* or *myl7*, genes with correlation coefficient > 0.7 across time are shown. Reanalysis of the published zebrafish bulk RNA-seq data was performed on the original dataset as published^38^.

### scRNA-seq and data processing

Transgenic zebrafish hearts at desired timepoints 1, 3 and 7 dpci were dissected, and the procedures for cardiac cell dissociation, viable sorting, transcript in vivo and sequencing follow our previous description^13^. Cells were harvested and libraries were generated by Single Cell Discoveries for 75-bp paired-end scRNA-seq using an Illumina NextSeq platform^77^. The resultant sequencing data were mapped against zebrafish reference genome (Zv9). Mitochondrial, oversequenced and ERCC spike-in mRNA reads were removed. Based on the distribution of the log_10_ total reads plotted against frequency, we introduced a cutoff at minimally 500 reads per cell to be included for further analysis. A total of 1,194 cells (280 1-dpi, 262 3-dpi and 652 7-dpi cells) were then processed in Seurat version 3.2.2 (ref. ^78^) with the following parameters: variable features = 500, dimensions = 8 and resolution = 0.7. Cells that were filtered with the above parameters were subjected to pseudotime analysis using Monocle 2 (ref. ^79^). Monocle 2 was used to identify genes differentially expressed over pseudotime (*P* < 0.05) and identified eight unsupervised clusters. For functional annotation of the genes, zebrafish gene IDs were converted to human ensemble IDs using Ensembl biomaRt^80,81^, and Gene Ontology term analysis was performed using DAVID^82^ and Metascape^26^. StemID2 (ref. ^83^) was used on the Seurat object, and values were projected in pseudotime with Monocle 2. Reanalysis of the published zebrafish scRNA-seq datasets was performed on the original datasets as published^13,27^.

scRNA and bulk RNA-seq data have been deposited at the GEO (Super Series GSE234990) and are publicly available as of the date of publication by accession numbers in the key resources table (Supplementary Table 21).

### Seahorse assay

The Seahorse XF Cell Mito Stress Test Kit (Agilent) was used to determine the OCR and ECAR of uninjured and 14-dpci zebrafish ventricles. The ventricles were positioned in an Islet Capture Microplate (Agilent, 101122-100) containing DMEM (Agilent, 1035755-100; 10 mM glucose, 1 mM pyruvate and 2 mM glutamine (pH 7.4)). Ten baseline (untreated), three oligomycin (MP Biomedicals) (50 μM), seven FCCP (Sigma-Aldrich) (30 μM) and nine rotenone (MP Biomedicals)/antimycin A (ChemCruz) (45 μM) measurements were acquired using the Seahorse XFe24 Analyzer (Agilent) at 28 °C. Seahorse traces were normalized against protein concentration using a Pierce BCA Assay Kit (Thermo Fisher Scientific). The OCR was defined as the average of the top three FCCP measurements.

### ^13^C metabolic analysis by nuclear magnetic resonance

To evaluate how glucose is metabolized, we injected 4 M ^13^C_6_ glucose (ChemCruz) in Milli-Q water intraperitoneally in wild-type zebrafish from the KCL strain at 7 dpci and then isolated and snap-froze the hearts 30 minutes after injection. To extract the metabolites, three replicates of 10 pooled ventricles were homogenized in 1 ml of pre-chilled 2:1 chloroform (Thermo Fisher Scientific): methanol (Sigma-Aldrich) extraction solution in a homogenizer (hard tissue grinding MK28; Precellys). The lysate was subsequently placed at 4 °C for 5 minutes before adding 400 μl of Milli-Q water, mixing thoroughly and centrifuging at 10,000*g* for 5 minutes at 4 °C. The aqueous phase containing the metabolites was isolated, freeze dried, reconstituted in deuterium oxide (D_2_O) and analyzed quantitatively using nuclear magnetic resonance (NMR) spectroscopy.

All NMR experiments were performed on a Bruker Avance NEO NMR spectrometer operating at a proton resonance frequency of 600 MHz and equipped with a BBO CryoProbe. Measurements were done at a temperature of 298 K. Data were acquired using the TopSpin 4.1.4 software package and analyzed using MestReNova (Mestrelab Research). Spectral annotations were performed according to the literature^84^.

### Scanning electron microscopy

Adult zebrafish WIK and SAT 7-dpci hearts were isolated and fixed at room temperature with a mixture of 2.5% glutaraldehyde/2% paraformaldehyde in 0.1 M sodium cacodylate buffer (pH 7.4). Hearts were then washed in 0.1 M PIPES (Thermo Fisher Scientific) and fixed with osmium tetroxide (2%) (Agar Scientific) and potassium ferricyanide (1.5%) (Merck) for 2 hours. After washing in buffer, the samples were treated with tannic acid followed by 2% osmium tetroxide and 1% uranyl acetate (Agar Scientific), respectively. After heavy metal staining, the samples were dehydrated through ethanol series, acetone and Durcupan/acetone gradient and embedded in Durcupan ACM resin (Sigma-Aldrich). Semi-thin sections were cut at 500 nm with a Leica Ultracut 7 ultramicrotome and mounted on a glass coverslip for counterstaining with lead citrate. Coverslips were finally mounted on stubs with conductive carbon adhesive tabs, and the heart tissue was coated with carbon (Quorum Technologies) for imaging.

### Imaging

Whole zebrafish images were taken using an iPhone X (Apple). Whole hearts were imaged with a Zeiss Stemi 2000-C stereomicroscope with a Zeiss Axiocam ERC5s camera (Carl Zeiss). (Immuno)histochemistry images of heart samples were acquired using a Nikon Eclipse Ci-L microscope with a Nikon DS-Fi3 camera and a Zeiss LSM 800 (Carl Zeiss). The scanning electron microscopy images were acquired at 10 kV with detector HDBSD (working distance 5.9 mm) using Zeiss Sigma 300 FEG-SEM.

### Quantification of stained sections

We used Fiji ImageJ software (https://imagej.net/Fiji)^85^ to quantify wound size and cellular foci of images. For AFOG-stained sections, we quantified wound size, including area and open wound length, on a minimum of three sections per heart with the largest wound. The wound size was presented as a percentage of wound area and length against the ventricular area and perimeter, respectively. Additionally, to characterize the regenerative state at 90 dpci, the hearts were visually categorized into three groups: (1) completely regenerated (total lack of wound throughout the heart), (2) incompletely regenerated with closed wound (only internal collagenous scar present) and (3) incompletely regenerated with open wound (absence of compact wall closure and/or fibrin deposition additionally to collagen deposits). The percentage of hearts in each category is presented per strain.

For proliferation measurements, we counted Mef2^+^PCNA^+^ double-labeled nuclei and those labeled only by Mef2^+^ or PCNA^+^MF20^+^ cells within the border zone, a 100-μm region adjacent to the wound border. The percentage of proliferating cardiomyocytes was obtained by dividing the number of PCNA^+^Mef2^+^ against the total Mef2^+^ nuclei or PCNA^+^ nuclei over the border zone area (pxl^2^). For embryonic myosin, we identified embcmhc signal in the border zone and normalized by inner wound length.

Amira software (Thermo Fisher Scientific) was used to quantify the area of the compact wall, trabecular muscle^86^ and atrium of the uninjured hearts. To correct for variation in atrium size due to differences in the size of the hearts, the atrial area was corrected by the total area of the heart.

### Statistics

Data analysis was performed blinded, and the data were plotted using GraphPad Prism version 9.1.0 (GraphPad Software). Animal numbers in each experiment and details on statistical tests are included in the figures or their legends. Data are presented as mean ± s.e.m. The box in the box plots indicates the 25th and 75th percentiles and the middle line the 50th percentile. The whiskers extend to the most extreme data points.

### Reporting summary

Further information on research design is available in the Nature Portfolio Reporting Summary linked to this article.

## Supplementary information

Supplementary Information Gating strategy for scRNA-seq. Dissociated injured hearts were FACS sorted with gates based on cell size and granularity to remove doublets, gating viable cells (DAPI^−^) and finally excited by 488 em525/50 and 532 em585/15 and to select the YFP^+^ population. **a**, Representative 3-dpci gating strategy. **b**, A non-fluorescent uninjured control was used to aid in selecting the correct cells.

Reporting Summary

Supplementary Tables 1–21 Supplementary Table 1: Gene Ontology term analysis of 7 dpci bulk RNA-seq genes negative correlating to 7 dpci wound length measurements (relating to Fig. 1g, top). Supplementary Table 2: Gene Ontology term analysis of 7 dpci bulk RNA-seq genes negative correlating to 90-dpci wound area measurements (relating to Fig. 1g, bottom). Supplementary Table 3: Upregulated genes from bulk RNA-seq in the 7 dpci NA compared to the 7 dpci SAT used for KEGG analysis (relating to Extended Data Fig. 3g). Supplementary Table 4: KEGG pathway enrichment analysis of the upregulated genes from bulk RNA-seq in the 7 dpci NA compared to the 7 dpci SAT (relating to Extended Data Fig. 3g). Supplementary Table 5: Gene Ontology term analysis of 7 dpci bulk RNA-seq genes positively correlating to 7 dpci border zone cardiomyocyte proliferation measurements (relating to Extended Data Fig. 4c). Supplementary Table 6: Gene Ontology term analysis of 7 dpci bulk RNA-seq genes positively correlating to 7 dpci embcmhc expression (relating to Fig. 4e, top). Supplementary Table 7: Gene Ontology term analysis of 7 dpci bulk RNA-seq genes positively correlating to 21 dpci embcmhc expression (relating to Fig. 4e, bottom). Supplementary Table 8: Cell counts and percentages contributing to Monocle 2 modules (relating to Extended Data Fig. 5f). Supplementary Table 9: Differentially expressed genes in pseudotime as presented in heatmap (relating to Fig. 5f). Supplementary Table 10: Gene Ontology term analysis of the differentially expressed genes of module 1 (relating to Fig. 5f). Supplementary Table 11: Gene Ontology term analysis of the differentially expressed genes of module 2 (relating to Fig. 5f). Supplementary Table 12: Gene Ontology term analysis of the differentially expressed genes of module 3 (relating to Fig. 5f). Supplementary Table 13: Gene Ontology term analysis of the differentially expressed genes of module 4 (relating to Fig. 5f). Supplementary Table 14: Gene Ontology term analysis of the differentially expressed genes of module 5 (relating to Fig. 5f). Supplementary Table 15: Gene Ontology term analysis of the differentially expressed genes of module 6 (relating to Fig. 5f). Supplementary Table 16: Gene Ontology term analysis of the differentially expressed genes of module 7 (relating to Fig. 5f). Supplementary Table 17: Gene Ontology term analysis of the differentially expressed genes of module 8 (relating to Fig. 5f). Supplementary Table 18: Gene Ontology term analysis of the upregulated genes from bulk RNA-seq in 7 dpci surface fish compared to 7 dpci Páchon cavefish (relating to Fig. 7a). Supplementary Table 19: Gene Ontology term analysis of the upregulated genes from bulk RNA-seq in 30 dpci surface fish compared to 30 dpci Páchon cavefish (relating to Fig. 7b). Supplementary Table 20: *P* values of differentially expressed Gene Ontology terms in surface fish compared to Páchon cavefish bulk RNA-seq throughout timepoints (relating to Extended Data Fig. 8c). Supplementary Table 21: Key resources table.

## Source data

Source Data Fig. 1 Statistical source data.

Source Data Fig. 2 Statistical source data.

Source Data Fig. 3 Statistical source data.

Source Data Fig. 4 Statistical source data.

Source Data Fig. 6 Statistical source data.

Source Data Extended Data Fig. 1 Statistical source data.

Source Data Extended Data Fig. 2 Statistical source data.

Source Data Extended Data Fig. 3 Statistical source data.

Source Data Extended Data Fig. 8 Statistical source data.

## Data Availability

The RNA-seq datasets generated in this study are deposited in the GEO repository under a SuperSeries with accession number GSE234990 (wild-type strains bulk RNA-seq: GSE234990; *Astyanax* bulk RNA-seq: GSE234989; scRNA-seq: GSE237276). Published datasets reanalyzed in this study come from the following studies: Hu et al.^27^ (GSE159032), Honkoop et al.^13^ (GSE139218) and Hill et al.^38^ (SRP117696). The raw data/measurements presented in this study are provided in the Source Data table. The materials used in this study are provided in the key resources table (Supplementary Table 21). Any further queries may be directed to the corresponding author, M.T.M.M. (mathilda.mommersteeg@dpag.ox.ac.uk). Source data are provided with this paper.

## Code availability

The code used to analyze the datasets is commercially available and is described and referenced in the Methods. Custom code relating to Extended Data Fig. 3 can be found at https://github.com/JasonJunYing/Oxphos_regeneration.
