# Supplementary material for: Oxidative phosphorylation is required for cardiomyocyte re-differentiation and long-term fish heart regeneration
Source: Nat Cardiovasc Res. 2025 Oct 1;4(10):1363–80. doi: 10.1038/s44161-025-00718-x (PMC12520976; doi:10.1038/s44161-025-00718-x)
Supplement: Supplementary file 1 — Gating strategy for scRNA-seq. Dissociated injured hearts were FACS sorted with gates based on cell size and granularity to remove doublets, gating viable cells (DAPI−) and finally excited by 488 em525/50 and 532 em585/15 and to select the YFP+ population. a, Representative 3-dpci gating strategy. b, A non-fluorescent uninjured control was used to aid in selecting the correct cells. [file 44161_2025_718_MOESM1_ESM.pdf]

nature cardiovascular research

Supplementary information https://doi.org/10.1038/s44161-025-00718-x

Oxidative phosphorylation is required for cardiomyocyte re-differentiation and long-term fish heart regeneration

In the format provided by the authors and unedited

a

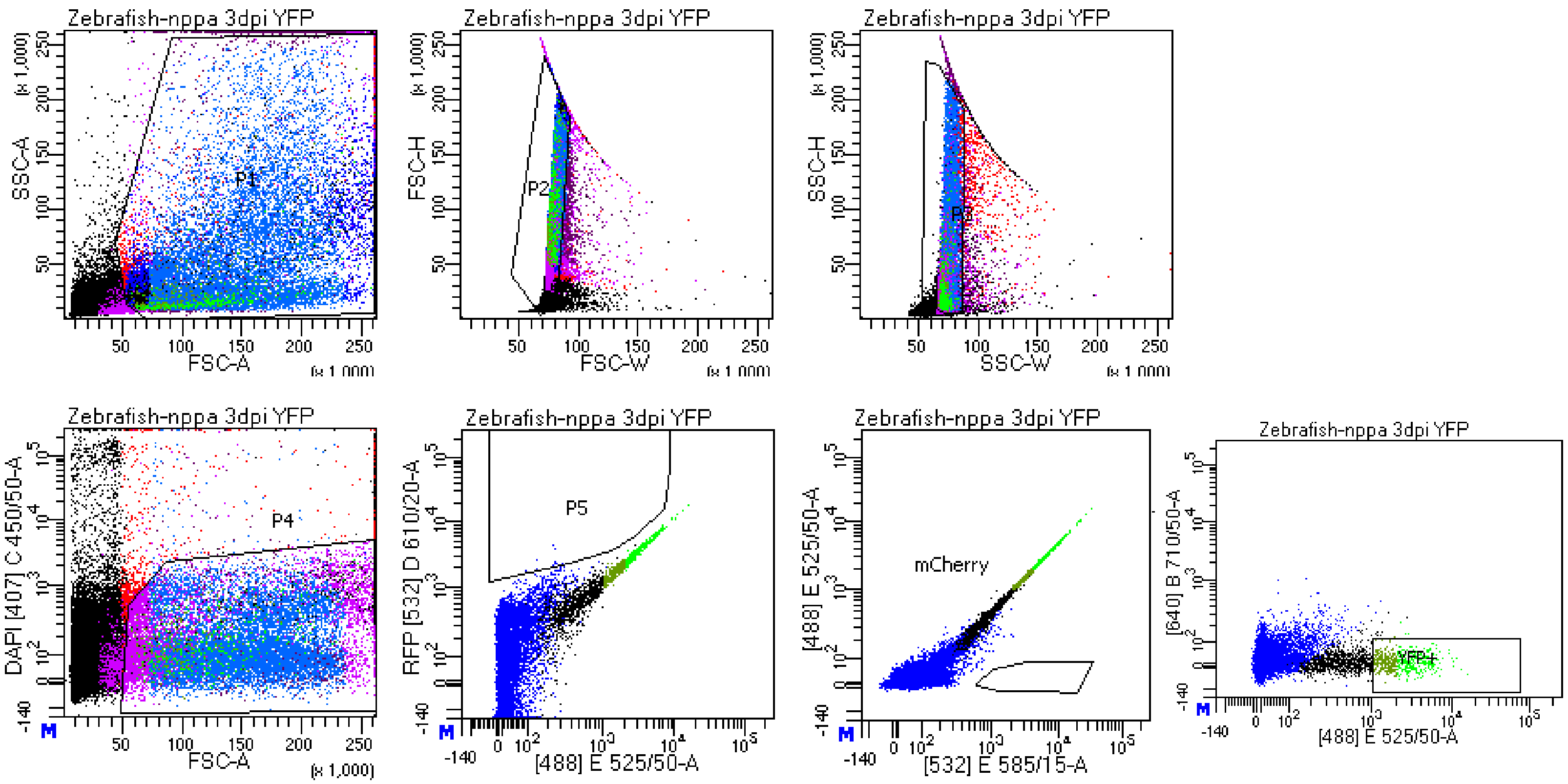
Zebrafish-nppa 3dpi YFP
SSC-A
FSC-A
(x 1,000)
P1
FSC-H
FSC-W
P2
SSC-H
SSC-W
DAPI [407] C 450/50-A
P4
RFP [532] D 610/20-A
[488] E 525/50-A
P5
mCherry
[532] E 585/15-A
[640] B 710/50-A
YFP+

b

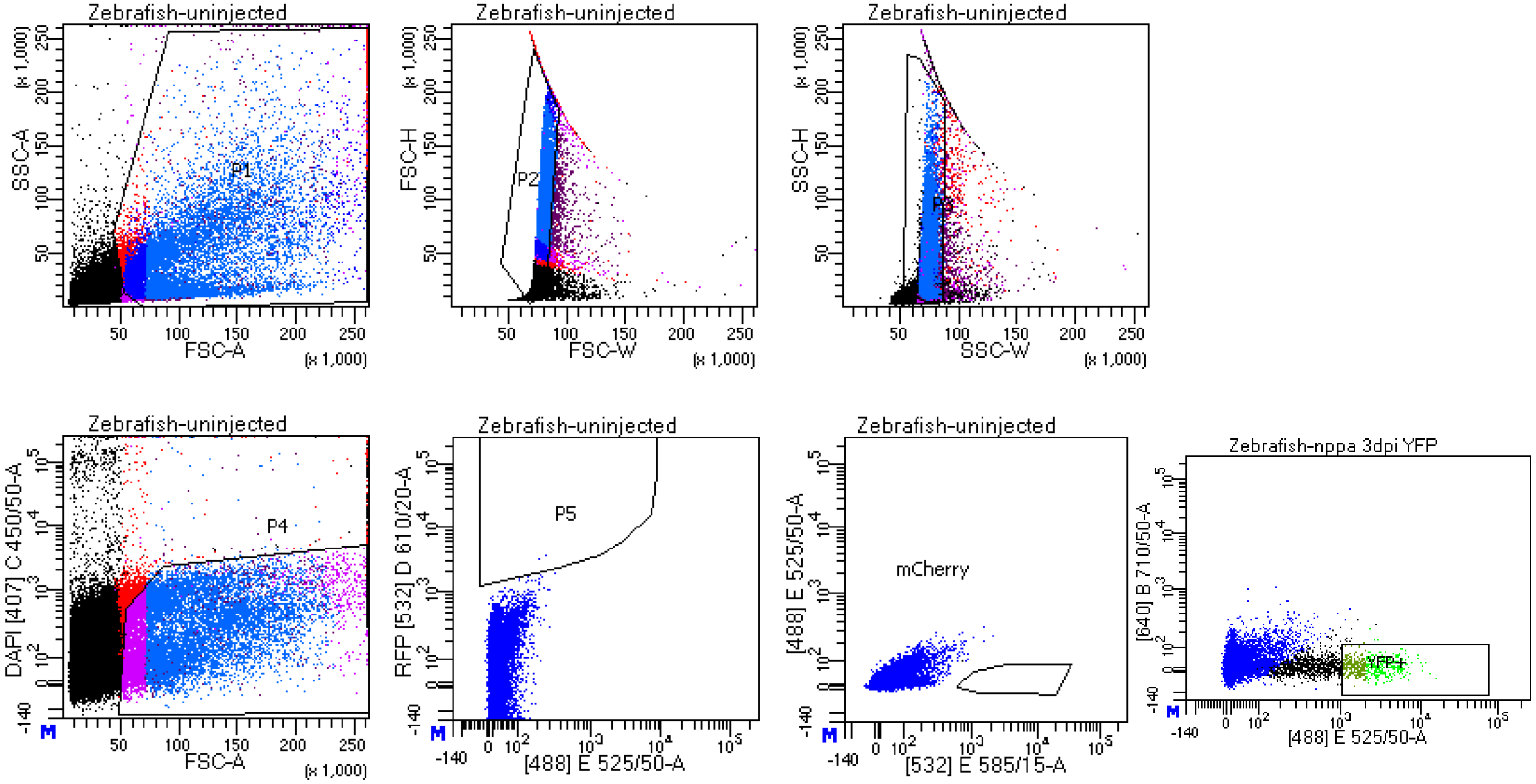
Zebrafish-uninjected
SSC-A
FSC-A
(x 1,000)
P1
FSC-H
FSC-W
P2
SSC-H
SSC-W
DAPI [407] C 450/50-A
P4
RFP [532] D 610/20-A
[488] E 525/50-A
P5
mCherry
[532] E 585/15-A
Zebrafish-nppa 3dpi YFP
[640] B 710/50-A
YFP+
