# Supplementary material for: Oxidative phosphorylation is required for cardiomyocyte re-differentiation and long-term fish heart regeneration
Source: Nat Cardiovasc Res. 2025 Oct 1;4(10):1363–80. doi: 10.1038/s44161-025-00718-x (PMC12520976; doi:10.1038/s44161-025-00718-x)
Supplement: Supplementary file 2 — Reporting Summary [file 44161_2025_718_MOESM2_ESM.pdf]

nature portfolio

Corresponding author(s): Mathilda TM Mommersteeg

Last updated by author(s): Jul 4, 2025

Reporting Summary

Nature Portfolio wishes to improve the reproducibility of the work that we publish. This form provides structure for consistency and transparency in reporting. For further information on Nature Portfolio policies, see our Editorial Policies and the Editorial Policy Checklist.

Statistics

For all statistical analyses, confirm that the following items are present in the figure legend, table legend, main text, or Methods section.

n/a	Confirmed	
☐	☒	The exact sample size (*n*) for each experimental group/condition, given as a discrete number and unit of measurement
☐	☒	A statement on whether measurements were taken from distinct samples or whether the same sample was measured repeatedly
☐	☒	The statistical test(s) used AND whether they are one- or two-sided<br>*Only common tests should be described solely by name; describe more complex techniques in the Methods section.*
☐	☒	A description of all covariates tested
☐	☒	A description of any assumptions or corrections, such as tests of normality and adjustment for multiple comparisons
☐	☒	A full description of the statistical parameters including central tendency (e.g. means) or other basic estimates (e.g. regression coefficient) AND variation (e.g. standard deviation) or associated estimates of uncertainty (e.g. confidence intervals)
☐	☒	For null hypothesis testing, the test statistic (e.g. *F*, *t*, *r*) with confidence intervals, effect sizes, degrees of freedom and *P* value noted<br>*Give P values as exact values whenever suitable.*
☒	☐	For Bayesian analysis, information on the choice of priors and Markov chain Monte Carlo settings
☒	☐	For hierarchical and complex designs, identification of the appropriate level for tests and full reporting of outcomes
☐	☒	Estimates of effect sizes (e.g. Cohen's *d*, Pearson's *r*), indicating how they were calculated

Our web collection on statistics for biologists contains articles on many of the points above.

Software and code

Policy information about availability of computer code

Data collection	Image acquisition was performed using the NIS software (version 5.01), SmartSEM software (version 7.02 service pack 3) and ZEN software (version14.0.2.201). NMR data were acquired using the Topspin 4.1.4 software. Seahorse measurements were acquired on the Wave software (version 2.6.1.56).
Data analysis	For the single-cell and bulk RNAseq analysis the following software were used: Zebrafish Genome assembly, Zebrafish gene annotation, Mexican tetra genome assembly, Mexican tetra gene annotation, Metascape, Kyoto Encyclopedia of Genes and Genomes (KEGG), EnrichR v.3.4.0, troppo v.0.0.7, RaceID2/StemID, Rstudio, R (v.4.0.1), Bcl2fastq (v2.20.0), Trim Galore! (v.0.6.4_dev), STAR (v.2.7.6a), DESeq2, RUVg, Benjamini-Hochberg algorith, Pheatmap (R package v1.0.8), Vennerable (R package v2.2/r79) gplots, Seurat (v3.2.2), Monocle2 (v2.18.0), DAVID, StemID2.<br>For the analysis of the NMR results, MestReNova (Mnova, v16.0.0) was used.<br>Image analysis was performed using ImageJ and Amira (v6.7.0).<br>Data visualisation and statistical analysis was performed using GraphPad Prism v9.10.

For manuscripts utilizing custom algorithms or software that are central to the research but not yet described in published literature, software must be made available to editors and reviewers. We strongly encourage code deposition in a community repository (e.g. GitHub). See the Nature Portfolio guidelines for submitting code & software for further information.

Data

Policy information about availability of data

All manuscripts must include a data availability statement. This statement should provide the following information, where applicable:

- Accession codes, unique identifiers, or web links for publicly available datasets
- A description of any restrictions on data availability
- For clinical datasets or third party data, please ensure that the statement adheres to our policy

The RNAseq datasets generated in this study are deposited in the GEO repository under a SuperSeries with accession number GSE234990 (wild-type strains bulk RNAseq GSE234990; Astyanax bulk RNAseq GSE234989; scRNAseq GSE237276). Published datasets re-analysed in this study come from the following studies: Hu et al. (GSE159032), Honkoop et al. (GSE139218) and Hill et al. (SRP117696). The raw data/measurements presented in this study are provided in the source data table. The materials used in this study are provided in the key resources table. Any further queries may be directed to the corresponding author, Mathilda Mommersteeg (mathilda.mommersteeg@dpag.ox.ac.uk).

Research involving human participants, their data, or biological material

Policy information about studies with human participants or human data. See also policy information about sex, gender (identity/presentation), and sexual orientation and race, ethnicity and racism.

Reporting on sex and gender	*Use the terms sex (biological attribute) and gender (shaped by social and cultural circumstances) carefully in order to avoid confusing both terms. Indicate if findings apply to only one sex or gender; describe whether sex and gender were considered in study design; whether sex and/or gender was determined based on self-reporting or assigned and methods used. Provide in the source data disaggregated sex and gender data, where this information has been collected, and if consent has been obtained for sharing of individual-level data; provide overall numbers in this Reporting Summary. Please state if this information has not been collected. Report sex- and gender-based analyses where performed, justify reasons for lack of sex- and gender-based analysis.*
Reporting on race, ethnicity, or other socially relevant groupings	*Please specify the socially constructed or socially relevant categorization variable(s) used in your manuscript and explain why they were used. Please note that such variables should not be used as proxies for other socially constructed/relevant variables (for example, race or ethnicity should not be used as a proxy for socioeconomic status). Provide clear definitions of the relevant terms used, how they were provided (by the participants/respondents, the researchers, or third parties), and the method(s) used to classify people into the different categories (e.g. self-report, census or administrative data, social media data, etc.) Please provide details about how you controlled for confounding variables in your analyses.*
Population characteristics	*Describe the covariate-relevant population characteristics of the human research participants (e.g. age, genotypic information, past and current diagnosis and treatment categories). If you filled out the behavioural & social sciences study design questions and have nothing to add here, write "See above."*
Recruitment	*Describe how participants were recruited. Outline any potential self-selection bias or other biases that may be present and how these are likely to impact results.*
Ethics oversight	*Identify the organization(s) that approved the study protocol.*

Note that full information on the approval of the study protocol must also be provided in the manuscript.

Field-specific reporting

Please select the one below that is the best fit for your research. If you are not sure, read the appropriate sections before making your selection.

☒ Life sciences ☐ Behavioural & social sciences ☐ Ecological, evolutionary & environmental sciences

For a reference copy of the document with all sections, see nature.com/documents/nr-reporting-summary-flat.pdf

Life sciences study design

All studies must disclose on these points even when the disclosure is negative.

Sample size	Sample sizes were determined based on previous experiments and power calculations. At least three biological replicates were performed for each experiment with the exception of the 1Dpci TU and SAT bulk RNAseq where only two biological replicates were used.
Data exclusions	No data was excluded from our analyses.
Replication	Each experiment includes measurements from several individuals which confirm our findings. Some of our experiments contain individuals from different generations (age matched) which show consistent results.
Randomization	No formal randomisation technique was used, but fish were allocated at random to the different experiments performed and the various timepoints for heart collection.

Blinding	Blinding was performed.

Reporting for specific materials, systems and methods

We require information from authors about some types of materials, experimental systems and methods used in many studies. Here, indicate whether each material, system or method listed is relevant to your study. If you are not sure if a list item applies to your research, read the appropriate section before selecting a response.

Materials & experimental systems

n/a	Involved in the study	
☐	☒	Antibodies
☒	☐	Eukaryotic cell lines
☒	☐	Palaeontology and archaeology
☐	☒	Animals and other organisms
☒	☐	Clinical data
☒	☐	Dual use research of concern
☒	☐	Plants

Methods

n/a	Involved in the study	
☒	☐	ChIP-seq
☒	☐	Flow cytometry
☒	☐	MRI-based neuroimaging

Antibodies

Antibodies used	Primary antibodies Mef2c (Biorbyt, orb576282), PCNA (Clone PC10, Dako, M0879), MF20 (DSHB, AB_2147781), GFP (abcam, ab13970) and embcmhc N2.261 (DSHB, AB_531790), and secondary antibodies, Alexa Fluor® 488 (Invitrogen, A11001, A21206 and A11039) and Alexa Fluor® 555 (Invitrogen, A31570), were prepared using TNB buffer at ratio of 1:200.
Validation	MF-20 (DSHB, AB_2147781) was validated in zebrafish by the manufacturer (https://dshb.biology.uiowa.edu/MF-20).<br>Mef2c (Biorbyt, orb576282) validated in human and mice and is predicted to react with zebrafish mef2c by the manufacturer (https://www.generon.co.uk/other-products-186/mef2c-antibody-716721721.html).<br>PCNA (Dako Cytomation, M0879) was validated in zebrafish by the manufacturer (https://www.labome.com/product/Dako/M0879.html).<br>anti-GFP is reactive to GFP of Aequorea victoria and is the most highly cited GFP antibody (>4700 times) according to the manufacturer (https://www.abcam.com/en-us/products/primary-antibodies/gfp-antibody-ab13970?srsltid=AfmBOorvAUnas-1dvfTvdVoRQ9WaAQNAPgo667JVNcLiTop2_aZFalep#)<br>N2.261 (DSHB, AB_531790) was validated in zebrafish by the manufacturer (https://dshb.biology.uiowa.edu/N2-261)<br>Secondary antibodies were validated by the manufacturer against the host of the primary antibodies, are highly cited and widely used.

Animals and other research organisms

Policy information about studies involving animals; ARRIVE guidelines recommended for reporting animal research, and Sex and Gender in Research

Laboratory animals	Danio rerio (zebrafish, strains: AB, NA, SAT, TL, TU, WIK and KCL) and Astyanax mexicanus (Mexican tetra, surface fish and Pachon cavefish) were used in this study. Experiments were performed on fish aged 0.5-2 years. Additionally, the zebrafish transgenic line TgBAC(nppa:mCitrine) (on a TL background) was used for the scRNAseq experiment, mdh1ab cOE and GFP cOE lines were created on the KCL background, and cardiodeleter, mdh1aa cKO and mdh1ab cKO strains were generated on the AB background . When analysing previously published datasets the strain of zebrafish is noted in the manuscript.
Wild animals	The study did not involve wild animals.
Reporting on sex	Both male and female fish were used in our experiments.
Field-collected samples	The study did not involve samples collected from the field.
Ethics oversight	All procedures involving animals at the University of Oxford, Boston College and Hubrecht Institute were approved by the local animal experiment committees and performed in compliance with animal welfare laws, guidelines and policies according to national and European law.

Note that full information on the approval of the study protocol must also be provided in the manuscript.

Plants

Seed stocks

Report on the source of all seed stocks or other plant material used. If applicable, state the seed stock centre and catalogue number. If plant specimens were collected from the field, describe the collection location, date and sampling procedures.

Novel plant genotypes

Describe the methods by which all novel plant genotypes were produced. This includes those generated by transgenic approaches, gene editing, chemical/radiation-based mutagenesis and hybridization. For transgenic lines, describe the transformation method, the number of independent lines analyzed and the generation upon which experiments were performed. For gene-edited lines, describe the editor used, the endogenous sequence targeted for editing, the targeting guide RNA sequence (if applicable) and how the editor was applied.

Authentication

Describe any authentication procedures for each seed stock used or novel genotype generated. Describe any experiments used to assess the effect of a mutation and, where applicable, how potential secondary effects (e.g. second site T-DNA insertions, mosiacism, off-target gene editing) were examined.
